# Validated thermal model for bacterial survival in fire-resistant self-healing concrete

**DOI:** 10.1038/s41598-025-13562-7

**Published:** 2025-08-01

**Authors:** Ajitanshu Vedrtnam, Kishor Kalauni, M. T. Palou

**Affiliations:** 1 Institute of Construction and Architecture, Slovak Academy of Science, Bratislava, 84503 Slovakia; 2 Department of Mechanical Engineering, Invertis University, Bareilly, 243001 UP India

**Keywords:** Self-healing concrete, Bacterial encapsulation, Fire resistance, Heat transfer modelling, Thermal durability, Engineering, Civil engineering

## Abstract

Bacteria-based self-healing concrete offers a sustainable solution to extend the service life of infrastructure by autonomously sealing cracks through microbial calcium carbonate precipitation. However, under fire conditions, the survival of encapsulated bacteria remains uncertain due to extreme temperatures that compromise biological activity and structural integrity. This study introduces a validated heat transfer model to estimate how long encapsulated bacteria can survive during fire exposure following ISO 834 conditions. The model incorporates radial heat diffusion, thermal properties of multi-layer encapsulation, and bacterial inactivation thresholds. Experimental data from our earlier study, including additional unpublished experimental insights, are used to validate the model across temperatures ranging from 200 °C to 800 °C. Simulations showed that carbon fiber-cement paste encapsulation can slow heat entry and help bacteria survive for nearly 20 h at 200 °C and about 4 h at 800 °C. In contrast, gelatin-based encapsulations degraded rapidly and failed to protect bacteria beyond 200 °C. Sensitivity analysis demonstrated that encapsulation thickness critically influences survival, with layers ≥ 1.75 mm providing significantly longer protection. This modelling framework, validated using prior experimental results on bacterial viability under fire exposure, provides a predictive basis for evaluating microbial survival in self-healing concrete systems employing multilayer encapsulation. The findings provide practical insights into optimizing encapsulation strategies to preserve bacterial functionality and enable post-fire self-healing in concrete structures.

## Introduction

Fire safety in cementitious materials is a critical focus area, driven by the growing frequency and severity of structural fires in modern infrastructure. Recent advancements in fire-resistant concrete design have aimed to enhance thermal stability, microstructural integrity, and mechanical performance under elevated temperatures^1–3^. Researchers have explored innovations such as fiber-reinforced composites, nano-engineered binders, hybrid cementitious systems, etc. to improve fire resistance and minimize spalling in load-bearing components^4–8^. However, a major limitation remains: conventional concrete lacks the capacity for autonomous repair after fire exposure. The thermal degradation of concrete leads to significant loss of mechanical strength, internal cracking, and spalling, primarily due to dehydration of calcium silicate hydrates, decomposition of calcium hydroxide, and pressure buildup from evaporating moisture^9–11^. To address these issues, considerable efforts have been made to enhance the thermal resilience of cementitious composites. These include the incorporation of polypropylene fibers to mitigate explosive spalling^12^, the use of supplementary cementitious materials (SCMs) such as fly ash and metakaolin to refine the microstructure^13^, and the development of geopolymer concretes that offer superior thermal stability^14^. Despite these developments, most fire-resistant strategies prioritize structural integrity during fire exposure, with little consideration for post-fire self-repair capabilities. This underscores the need for multifunctional materials that not only withstand fire but also restore their performance autonomously after thermal events.

In this context, bacteria-based self-healing concrete has emerged as a promising and sustainable technology. In recent years, self-healing concrete technologies have gained significant attention due to their potential to improve the longevity and durability of structures. One of the most promising approaches involves embedding bacteria and calcium lactate within concrete to enable self-healing by biogenic mineral precipitation^15,16^. However, for such bacteria-based self-healing concrete to be viable in real-world applications, it must withstand extreme environmental conditions, including high temperatures during fire events^17,18^. While microbial self-healing concrete has been widely studied in standard environmental conditions, its viability in high-temperature applications, such as fire-exposed structures, remains largely unexplored. Fires can cause thermal damage and microstructural changes in concrete, making it difficult for embedded bacteria to survive. At temperatures above 100 °C, free water evaporates, reducing bacterial hydration and metabolic function. Beyond 200 °C, bacterial spores experience protein denaturation and cytoplasmic degradation, leading to irreversible inactivation^19^. By 400–600 °C, dehydration of calcium silicate hydrates and decomposition of calcium hydroxide result in severe mechanical deterioration of the concrete matrix, further compromising microbial viability^20–23^. Additionally, pore expansion and thermal stress-induced cracking may disrupt encapsulation integrity, affecting the controlled release and activation of bacteria^15^. Improving thermal protection for bacteria is essential to create fire-resistant self-healing concrete.

While concrete itself is known for its fire resistance^24^, the survival of bacteria embedded within remains a critical barrier to achieving reliable post-fire healing. Recent studies have explored encapsulation techniques to improve bacterial survival under elevated temperatures^24–27^. Silica-based and polymer coatings have been investigated as protective barriers^28,29^ but many suffer from chemical instability or degradation in alkaline environments^30^. Mineral-based encapsulation using expanded clay or lightweight aggregates has also been studied for thermal insulation^31,32^; however, these may hinder bacterial activation due to low permeability^33^. A robust encapsulation system must therefore balance thermal shielding with controlled bacterial release under post-crack moisture exposure.

Our recent experimental work^34^ introduced novel encapsulation systems such as carbon fiber bacteria balls (CFBB) and gelatin capsules to assess bacterial survival in concrete subjected to fire. Fire tests confirmed partial survival and post-fire self-healing functionality in optimized systems. However, such studies relied heavily on experimental observations without predictive tools to anticipate survival thresholds under varying fire conditions.

Parallel to material innovations, thermal behavior modeling has advanced significantly. State-of-the-art simulations now address coupled thermo-mechanical and hygro-thermal phenomena, enabling prediction of damage progression and post-fire residual strength in concrete structures^35–37^. Several studies have addressed thermal modelling in fire-exposed concrete using finite element methods and analytical conduction models, most focus exclusively on temperature evolution and structural integrity. Banerjee^38^ reviewed thermal models in fire design but did not account for microbial aspects. Similarly, Tahersima and Tikalsky^39^ simulated hydration heat and thermal propagation in slabs but without any biological integration. In contrast, bio-concrete studies have evaluated bacterial viability experimentally after thermal exposure but lack predictive frameworks. Pungrasmi et al.^26^ explored encapsulation methods for microbial survival, while Vedrtnam et al.^34^ reported post-fire bacterial viability based on laboratory data. However, these efforts do not incorporate microbial inactivation thresholds into heat transfer simulations. Most recently, Javeed et al.^40^ reviewed microbial self-healing in concrete but noted the absence of models coupling thermal damage with bacterial survival. Our study advances this field by integrating microbial lethality kinetics into a multilayer radial heat transfer model. Specifically, we simulate transient temperature profiles across carbon fiber, cement paste, and concrete layers and predict bacterial survival time based on the critical 70 °C threshold for Bacillus subtilis spores. This approach enables not only survival zone prediction but also encapsulation optimization.

The present study addresses this gap by developing a validated transient heat transfer model to predict bacterial viability based on fire exposure time, encapsulation configuration, and material properties. Unlike conventional numerical models, which focus only on heat distribution^41,42^, our approach integrates bacterial inactivation kinetics with multi-layer encapsulation geometries, allowing simulation of survival zones and critical exposure durations. This approach builds on thermal modelling principles established in concrete fire safety research and aligns with recent efforts to model microbial processes in construction materials^40^. Unlike conventional finite element models that primarily simulate temperature distributions in structural materials, the present study advances existing approaches by explicitly integrating bacterial inactivation kinetics into a multilayer radial heat transfer model. This simulation framework resolves transient heat conduction across carbon fiber, cement paste, and concrete layers, while incorporating a biologically relevant survival threshold (70 °C) to predict bacterial viability over time. By capturing the spatial evolution of survival zones and coupling thermal diffusion with microbial behaviour, the model offers a functionally relevant approach for evaluating bacterial protection under fire exposure. To our knowledge, such integration of microbial viability within thermal models for encapsulated systems has not been previously reported. To assess model fidelity, simulation outputs are compared with experimental results reported by Vedrtnam et al.^34^, involving similar encapsulation materials and fire-exposure conditions. This serves as an initial yet rigorous validation step, demonstrating the model’s ability to predict bacterial survival behaviour under fire-induced thermal conditions within the tested configuration. While promising, broader experimental datasets will be needed to fully establish the model’s generalizability across material systems and fire scenarios.

## Key research objectives

This study explores the thermal resilience of bacteria-based self-healing concrete under fire conditions by developing a predictive framework that integrates numerical modelling with experimental validation. The research focuses on four primary objectives:

1. **To investigate heat transfer dynamics** within a multilayer encapsulation system designed to protect bacteria embedded in concrete. The system includes carbon fiber, cement paste, and an outer concrete layer. The study analyses how these layers delay temperature rise and protect bacteria from lethal exposure, particularly during ISO 834 standard fire scenarios.
2. **To quantify bacterial survival times** across a range of furnace temperatures (200 °C to 800 °C). This parametric analysis estimates the duration required for encapsulated bacteria to reach 70 °C—a critical inactivation threshold—providing insight into thermal tolerance and encapsulation performance.
3. **To develop a validated predictive model** for simulating thermal behaviour within encapsulated systems. Using heat transfer formulations—including the Lumped Capacitance Model and Fourier’s Law—the study constructs a numerical model that simulates radial heat diffusion and inactivation kinetics. The model is validated using previously reported experimental data^34^ and offers a generalized framework applicable to a variety of encapsulation strategies and fire conditions.
4. **To assess the engineering implications** of bacterial survival for self-healing concrete in fire-prone environments. By exploring encapsulation geometry, material choices, and thermal shielding effectiveness, the study proposes design guidelines to enhance fire resilience and extend the post-fire healing potential of concrete structures.

This research advances the field by shifting from empirical post-fire evaluation toward a predictive, design-oriented approach. If the modelling framework confirms viable bacterial activity after fire exposure, it could inform the development of self-repairing concrete systems with reduced post-fire maintenance costs and improved durability. Conversely, where survival thresholds are unmet, the findings will highlight necessary encapsulation upgrades. Ultimately, the study contributes to the development of next-generation fire-resistant self-healing concrete for use in tunnels, high-rise buildings, transport hubs, and other high-risk infrastructure.

## Methodology

To evaluate the thermal survival time of bacteria encapsulated within a protective multi-layer system, a computational heat transfer model, validated by experiment, was developed. The study simulates heat transfer through encapsulated bacteria in a concrete-embedded system exposed to different furnace temperatures (200 °C, 400 °C, 600 °C, and 800 °C) to determine how long the bacteria can survive before reaching 70 °C, a temperature beyond which bacterial survival is questionable.

### Description of the multi-layer encapsulation system

Bacteria and calcium lactate are enclosed in a three-layer system that slows heat and helps bacterial survival at elevated temperatures. The first layer, the inner encapsulation layer, consists of a 1 mm thick carbon fiber shell. This layer provides primary mechanical and thermal protection to the bacteria, ensuring that the encapsulated system remains intact under high-temperature conditions. The thermal properties of carbon fiber include a thermal conductivity of 10 W/m·K, a density of 1600 kg/m^3^, and a specific heat capacity of 900 J/kg·K^43^, all of which contribute to its role in delaying heat transfer to the bacteria. The second layer is the middle coating layer, made of 1 mm thick cement paste. This additional layer serves as a secondary thermal buffer, further delaying the penetration of heat into the core. The thermal properties of cement paste include a thermal conductivity of 1.0 W/m·K, a density of 2200 kg/m^3^, and a specific heat capacity of 900 J/kg·K^44^, which enhance its ability to slow down thermal diffusion and provide extra protection to the bacteria. The final layer, the outer protective layer, is composed of a 150 mm × 150 mm × 150 mm cubes concrete block. This layer acts as the bulk insulation, providing significant thermal resistance to the system. The thermal properties of concrete include a thermal conductivity of 1.4 W/m·K, a density of 2400 kg/m^3^, and a specific heat capacity of 880 J/kg·K^45^, making it effective at slowing heat transfer to the bacteria. The encapsulated system is assumed to be placed at the center of the concrete block during modelling, ensuring uniform exposure to heat from all directions and thereby protecting the bacteria from rapid temperature fluctuations during fire exposure.

Bacterial survival was tested by heating the encapsulated system at four furnace temperatures to simulate different fire conditions. The test conditions were established across four cases, each set at a different temperature: Case 1 at 200 °C, Case 2 at 400 °C, Case 3 at 600 °C, and Case 4 at 800 °C. To simulate realistic fire exposure, the ISO 834 fire curve was followed, which mirrors the standard temperature-time progression used in fire resistance testing within the construction industry. One of the objectives of the model was to determine the time required for the bacteria to reach 70 °C inside the encapsulation at each of these furnace temperatures. This approach allowed for a consistent and reliable evaluation of bacterial survival in response to fire exposure, providing critical data for optimizing bacterial encapsulation strategies under high-temperature conditions.

### Heat transfer modelling approach

A combined analytical-numerical approach was used to model transient heat conduction within the encapsulated concrete system. The system was idealized as a multilayer sphere subjected to uniform external heating, allowing temperature evolution to be tracked from the concrete surface to the bacterial core.

#### Lumped capacitance model (LCM) for encapsulation system

The present study employs a three-dimensional finite difference method (FDM) to solve the transient heat conduction equations across multilayer encapsulation geometries. Since the carbon fiber and cement layers are thin (1 mm each), the Lumped Capacitance Model (LCM) was used to estimate temperature changes inside the encapsulation. The temperature evolution inside the encapsulated system was computed using the transient heat transfer Eq. (1).

1 $$\:T\left(t\right)={T}_{\infty\:}-({T}_{\infty\:}-{T}_{0}){e}^{-hAt/m{c}_{p}}$$

where, *T(t)* is temperature inside the encapsulation at time *t*, *T*_*∞*_ is furnace temperature (varied between 200 °C and 800 °C), *T*_*0*_ = 23 °C (initial ambient temperature), *h* is 25 W/m^2^⋅Kh (convective heat transfer coefficient), *A* = surface area of encapsulation (assumed as a sphere), *m* is mass of encapsulation, cement paste and concrete, and *c*_*p*_ is effective heat capacity of the multi-layer system.

This equation was used to predict temperature rise inside the encapsulation over time. Since the concrete block is large, heat transfer within it was modelled using Fourier’s Law for transient heat conduction (Eq. (2)).

2 $$\:q=k\varDelta\:T/L$$

where, *q* is heat transfer rate, *k* = thermal conductivity of concrete (1.4 W/m·K), *ΔT* is temperature difference between the furnace and encapsulated bacteria, and *L* is distance from concrete surface to encapsulated bacteria.

Heat conduction was solved numerically to estimate how long it takes for the bacteria to reach 70 °C inside the concrete block. The generalized transient heat conduction equation in three-dimensional form for multilayer encapsulation, assuming that the temperature varies with time and in all three spatial dimensions, is given by Eq. (3).

3 $$\:\frac{\partial\:T\:(x,y,z,t)}{\partial\:t}=\alpha\:\left(\frac{{\partial\:}^{2}T(x,y,z,t)}{\partial\:{x}^{2}}+\frac{{\partial\:}^{2}T(x,y,z,t)}{\partial\:{y}^{2}}+\frac{{\partial\:}^{2}T(x,y,z,t)}{\partial\:{z}^{2}}\right)$$

where, *T(x*,* y*,*z*,* t)* is the temperature as a function of position in the *x*, *y*, and *z* directions and time *t*, and *α* is the thermal diffusivity of the material.

For a multilayer encapsulation system, heat transfer occurs through each layer, necessitating the individual resolution of the heat conduction equation for the carbon fiber (inner layer), cement paste (middle layer), and concrete (outer layer). The thermal properties—thermal conductivity, density, and specific heat capacity—of each material are incorporated into their respective layers. The temperature evolution within each layer is governed by the heat conduction equation, with boundary conditions at each interface solved sequentially. Using these established conditions, the time-dependent temperature rise within the encapsulation system was simulated. This modelling allows us to determine the critical time at which the internal temperature of the bacteria reaches the survival threshold of 70 °C. Thus, customised governing equations for the multilayer encapsulation system are given by Eqs. (4–6).

Layer 1 (Carbon Fiber):

4 $$\:\frac{\partial\:{T}_{1}\:(x,y,z,t)}{\partial\:t}={\alpha\:}_{1}\left(\frac{{\partial\:}^{2}{T}_{1}(x,y,z,t)}{\partial\:{x}^{2}}+\frac{{\partial\:}^{2}{T}_{1}(x,y,z,t)}{\partial\:{y}^{2}}+\frac{{\partial\:}^{2}{T}_{1}(x,y,z,t)}{\partial\:{z}^{2}}\right)$$

Layer 2 (Cement Paste):

5 $$\:\frac{\partial\:{T}_{2}\:(x,y,z,t)}{\partial\:t}={\alpha\:}_{2}\left(\frac{{\partial\:}^{2}{T}_{2}(x,y,z,t)}{\partial\:{x}^{2}}+\frac{{\partial\:}^{2}{T}_{2}(x,y,z,t)}{\partial\:{y}^{2}}+\frac{{\partial\:}^{2}{T}_{2}(x,y,z,t)}{\partial\:{z}^{2}}\right)$$

Layer 3 (Concrete):

6 $$\:\frac{\partial\:{T}_{3}\:(x,y,z,t)}{\partial\:t}={\alpha\:}_{3}\left(\frac{{\partial\:}^{2}{T}_{3}(x,y,z,t)}{\partial\:{x}^{2}}+\frac{{\partial\:}^{2}{T}_{3}(x,y,z,t)}{\partial\:{y}^{2}}+\frac{{\partial\:}^{2}{T}_{3}(x,y,z,t)}{\partial\:{z}^{2}}\right)$$

For this multilayer system, appropriate boundary conditions need to be applied at the interfaces between layers and at the outer surface of the encapsulation system (Eqs. 7–9).

- At the interface between carbon fiber and cement paste, the temperature gradient and heat flux must be continuous across the interface.

7 $$\:\frac{\partial\:{T}_{1}\:}{\partial\:x}=\frac{\partial\:{T}_{2}\:}{\partial\:x},\:\:\frac{\partial\:{T}_{1}\:}{\partial\:y}=\frac{\partial\:{T}_{2}\:}{\partial\:y},\:\:\frac{\partial\:{T}_{1}\:}{\partial\:z}=\frac{\partial\:{T}_{2}\:}{\partial\:z}$$

- At the interface between cement paste and concrete, the temperature gradient and heat flux must be continuous across the interface.

8 $$\:\frac{\partial\:{T}_{2}\:}{\partial\:x}=\frac{\partial\:{T}_{3}\:}{\partial\:x},\:\:\frac{\partial\:{T}_{2}\:}{\partial\:y}=\frac{\partial\:{T}_{3}\:}{\partial\:y},\:\:\frac{\partial\:{T}_{2}\:}{\partial\:z}=\frac{\partial\:{T}_{3}\:}{\partial\:z}$$

- At the outer surface of the concrete layer, the heat flux due to convection to the surrounding environment at outer surface.

9 $$\:\frac{\partial\:{T}_{2}\:}{\partial\:x}=\frac{\partial\:{T}_{3}\:}{\partial\:x},\:\:\frac{\partial\:{T}_{2}\:}{\partial\:y}=\frac{\partial\:{T}_{3}\:}{\partial\:y},\:\:\frac{\partial\:{T}_{2}\:}{\partial\:z}=\frac{\partial\:{T}_{3}\:}{\partial\:z}$$

These governing equations, combined with the boundary conditions, are solved using numerical using Finite Difference Method (FDM). By discretizing the spatial and time dimensions, the temperature profile inside the encapsulation system can be computed over time, providing insights into bacterial survival and optimizing encapsulation designs under fire conditions. This integrated framework allowed prediction of bacterial viability as a function of furnace temperature, exposure time, and encapsulation geometry, enabling optimization of fire-resistant self-healing concrete systems.

#### Computational implementation

The heat transfer equations within the multilayer encapsulation system were solved through Python-based numerical simulations, executed in structured phases. Initially, the material properties for each layer—thermal conductivity, specific heat capacity, and density of the carbon fiber, cement paste, and concrete—were specified. Following this, the initial and boundary conditions were set, with the encapsulation’s starting temperature at 23 °C (room temperature), and the furnace temperatures aligned with the test conditions at 200 °C, 400 °C, 600 °C, and 800 °C. The simulations employed the Linear Crank-Nicolson Method (LCM) to model the temperature rise in the encapsulation and Fourier’s Law to simulate heat conduction through each layer. This approach allowed the tracking of temperature evolution over time, pinpointing the critical durations at which bacterial temperatures reached survival thresholds under each condition. The outcomes were graphically represented, illustrating the temperature-time profiles for each test scenario and effectively demonstrating the thermal effects on bacterial viability at varied temperatures.

For each furnace temperature (200 °C, 400 °C, 600 °C, and 800 °C), the model tracked the temperature evolution at the center of the encapsulated region using the transient heat conduction equations. The bacterial survival time was defined as the time taken for this central temperature to reach 70 °C, the critical inactivation threshold for *Bacillus subtilis* spores. The simulation ran until this condition was met, and the corresponding time point was recorded as the survival time. This procedure was applied uniformly to ensure comparability across all fire exposure scenarios and forms the basis for the survival time data.

To numerically solve the heat conduction equation, the FDM is employed. The computational domain is divided into a uniform 3D grid, where temperature values are updated iteratively at each time step. The grid points, denoted as (i, j, k), correspond to discrete locations along the X, Y, and Z axes. At each time step, temperature updates are computed using an explicit finite difference scheme, with the time derivative approximated by forward differences.

#### Assumptions and limitations

To simplify the analysis of the system, certain assumptions were made. Notably, the model does not account for thermal cracking or explosive spalling, which can significantly influence heat transfer pathways and compromise encapsulation integrity. Future modelling efforts should incorporate thermo-mechanical coupling to simulate crack propagation and damage evolution under high-temperature conditions. Additionally, the thermal properties of materials are treated as constant, despite their known variability with temperature and microstructural degradation. Incorporating temperature-dependent thermal properties and evolving porosity could improve accuracy. The model also omits hygrothermal effects such as moisture migration and vapor pressure build-up, which are critical in real fire scenarios; integrating a multiphysics approach could address these limitations. Furthermore, bacterial inactivation is simplified using a fixed 70 °C threshold, whereas in reality, inactivation follows a time-temperature-dependent kinetic process. Advanced microbial models based on Arrhenius or D-value kinetics could better represent this behavior. Lastly, structural deterioration such as interfacial delamination or encapsulation breakdown is not dynamically linked to thermal exposure in the current framework. Future work should integrate these structural effects to enable a more comprehensive simulation of bacterial survival and self-healing viability under fire exposure.First, the encapsulation layers, made of carbon fiber and cement paste, are presumed to maintain a uniform temperature distribution across their thickness. Additionally, the furnace temperature is considered steady and uniform following the initial heating phase. The model also does not consider moisture loss or the chemical decomposition of concrete at elevated temperatures. The model also does not account for temperature-induced micro- and meso-scale damage in cementitious materials, such as increased porosity, microcrack propagation, and void coalescence. These structural changes, as demonstrated by CT imaging studies^46^, can significantly influence thermal diffusivity by enhancing pore connectivity at elevated temperatures. Although our present model assumes homogenous and intact thermal properties for clarity and computational efficiency, future work will incorporate evolving thermal behavior linked to microstructural degradation under fire exposure. Further, it is assumed that the bacteria within the encapsulation system remain dormant until reaching the lethal temperature threshold of 70 °C.

In addition, the model assumes perfect thermal continuity at material interfaces—specifically between carbon fiber, cement paste, and the surrounding concrete. This simplification neglects the potential thermal resistance introduced by interfacial transition zones (ITZs), which may exhibit distinct microstructural characteristics affecting localized heat transfer. Although the ITZ thickness is typically small (~ 10–50 μm), its influence may become significant under steep temperature gradients or extended fire exposure. This is acknowledged as a limitation of the present model, and future work may incorporate thermal contact conductance or multi-scale modeling to account for such effects.

### Experimental tests on thermal viability

To support the development and validation of the proposed heat transfer model, experimental results from our previous study^34^ were used to assess the thermal protection afforded by bacterial encapsulation in self-healing concrete. That study evaluated the survival of *Bacillus subtilis* spores encapsulated within carbon fiber shells coated with cement paste, which were embedded in concrete specimens and exposed to elevated furnace temperatures.

Concrete samples were prepared using encapsulated self-healing agents and subjected to controlled heating at 200 °C, 400 °C, 600 °C, and 800 °C, each for one hour. The temperature profiles followed a modified ISO 834 fire curve to replicate realistic fire conditions. Post-heating, mechanical testing and microbial viability assays were conducted to determine the extent of bacterial survival and any retained self-healing potential. Bacterial viability after fire exposure was assessed using the standard spread plate method. Immediately after controlled cooling, the encapsulated core material was retrieved using sterile tools, suspended in sterile phosphate-buffered saline (PBS), and vortexed for 1 min to ensure homogenization. Serial dilutions were performed, and 100 µL aliquots were plated in duplicate onto nutrient agar plates. Plates were incubated at 37 °C for 48 h, and colony-forming units (CFUs) were counted manually. Control specimens (not subjected to fire) were processed identically and served as 100% viability benchmarks. Each experimental condition was tested in biological triplicates (*n* = 3), and viability was expressed as mean ± standard deviation (SD)^34^. Control (non-heated) specimens were prepared identically and processed in parallel with the fire-exposed samples. These served as 100% viability reference points for calculating relative bacterial survival. All viability data are expressed as percentages normalized to these non-heated controls. The results demonstrated that encapsulation using carbon fiber and cement paste significantly improved bacterial resilience at temperatures up to 600 °C, with partial metabolic activity still detected after exposure.

These experimental observations provided critical benchmarks for calibrating the predictive model developed in this study. In particular, the survival durations at different thermal loads were used to validate simulated inactivation thresholds and heat penetration behaviour across multilayer encapsulation systems. By integrating previously validated experimental findings into the current numerical framework, this work builds a more robust and generalizable understanding of bacterial thermal survivability in fire-prone concrete structures.

### Sample Preparation

**Fig. 1:**
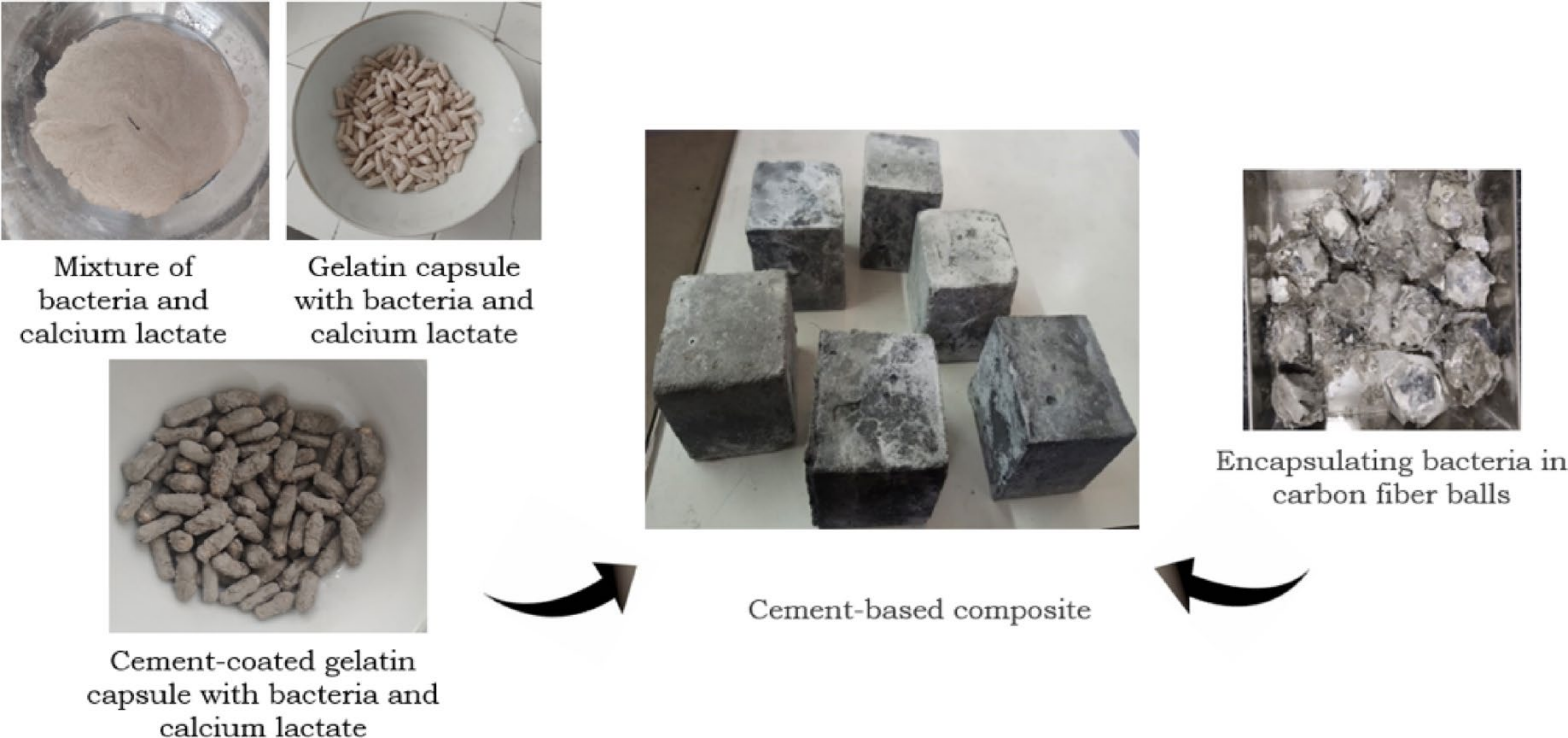
Self-healing concrete encapsulated in CFBB and gelatin capsules.

Concrete specimens used to validate the proposed heat transfer model were prepared using an M40 mix design, selected for its high strength and durability. Ordinary Portland cement (OPC), fine and coarse aggregates, and water were combined in standard proportions. The self-healing system incorporated *Bacillus subtilis* spores encapsulated in two forms: carbon fiber bacteria balls (CFBB) and gelatin capsules, both coated with a 1 mm thick cement paste layer for enhanced thermal protection (Fig. 1). These encapsulated agents were uniformly distributed within the concrete matrix at volume fractions of 10%, 15%, and 20%, alongside calcium lactate as the nutrient source.

CFBB was selected for its excellent thermal resistance (~ 10 W/m·K), while gelatin capsules served as a comparative encapsulation strategy with lower thermal durability. The encapsulated agents were incorporated in powdered form, maintaining bacterial concentrations of 10^6^ to 10^8^ cells/mL—consistent with values shown to be effective for self-healing performance^34^. The concrete specimens were cast in 150 mm × 150 mm × 150 mm cubes and cured for 28 days before fire exposure. A small section of CFBB-containing concrete was also prepared for SEM analysis following standard sputter-coating and imaging procedures to observe post-fire microstructural changes.

These materials and mix designs have been previously validated for self-healing and post-fire survival performance^34^. In the present study, they provide a foundation for calibrating and validating thermal diffusion and bacterial survival models under controlled fire exposure conditions.

### Furnace exposure and fire testing

**Fig. 2:**
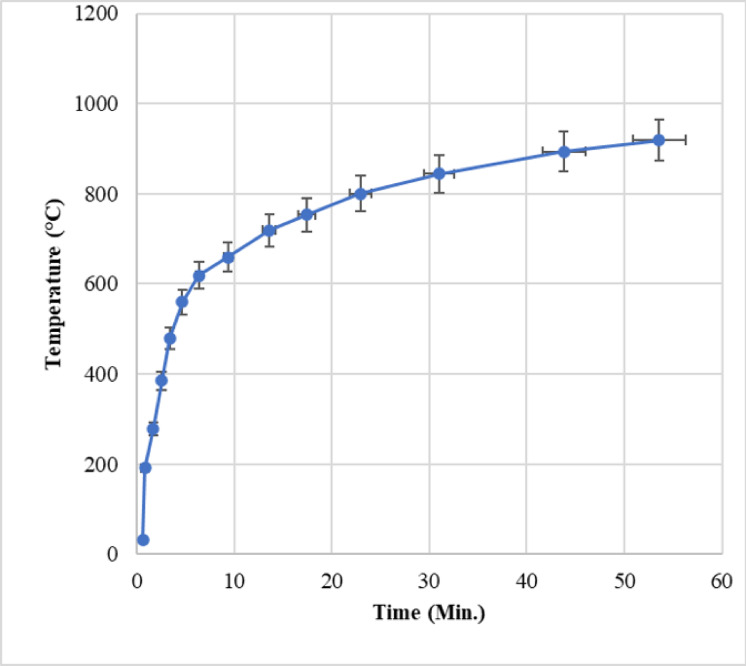
Modified ISO 834 standard temperature-time curve.

To evaluate the thermal performance of encapsulated bacteria and calcium lactate within the concrete matrix, 150 mm × 150 mm × 150 mm concrete specimens were subjected to controlled furnace exposure. The testing followed a modified ISO 834 standard temperature–time curve (Fig. 2) to simulate realistic structural fire conditions, including heat progression and intensity. These experiments, previously described in detail^34^, provide the empirical basis for validating the predictive thermal model developed in this study.

In this study, thermal exposure of specimens was performed using a controlled electric furnace, following a modified ISO 834 standard fire curve. The modification involved following the ISO 834 temperature-time trajectory up to predefined target temperatures of 200 °C, 400 °C, 600 °C, or 800 °C, after which the temperature was held constant for the remainder of the 1-hour exposure period. This approach enabled simulation of different severities of fire exposure under regulated conditions. Although our earlier study^34^ employed both open-flame and furnace-based heating, only the furnace-based tests conducted under these temperature-limited ISO 834 conditions were used in the present modeling and validation. To ensure accurate internal temperature readings, K-type thermocouples were embedded within the concrete specimens at multiple depths, including at the core and near-surface regions. These thermocouples were fully encased within the matrix, insulated from the furnace atmosphere, and unaffected by external flame or radiative interference. The recorded data therefore represent the internal thermal environment relevant to bacterial viability and modeling input.

The furnace tests were conducted at four temperature levels:

- 200 °C, representing moderate early-stage fire exposure,
- 400 °C, simulating sustained fire in structural components,
- 600 °C, indicative of flashover-level conditions, and.
- 800 °C, mimicking near-full engulfment scenarios.

Each specimen was centrally positioned in the furnace and exposed for one hour. The heating profile was carefully regulated to ensure gradual temperature rise, avoiding thermal shock. Thermocouples embedded at multiple depths captured surface and core temperatures, enabling analysis of heat transfer dynamics and encapsulation performance. The critical objective was to determine the time taken for the encapsulated region to reach 70 °C, the threshold beyond which *Bacillus subtilis* viability significantly diminishes.

Following thermal exposure, specimens were left to cool gradually within the furnace over 24 h to avoid cracking and preserve structural integrity. Post-cooling, mechanical and microbial analyses were performed to evaluate the extent of thermal degradation, bacterial survival, and potential for post-fire self-healing. These results served as key validation data for the heat transfer simulations, particularly in benchmarking predicted survival times and verifying modelled thermal gradients within multilayer encapsulation systems.

## Results and discussion

### Numerical simulation analysis of bacterial survival in encapsulated concrete

The numerical simulations provided insights into how long bacteria can survive when encapsulated inside a multi-layer protective system embedded in concrete, subjected to varying furnace temperatures. The primary output of the analysis was the time required for the encapsulated bacteria to reach 70 °C, beyond which bacterial survival is unlikely. The results demonstrate that higher furnace temperatures significantly reduce bacterial survival time due to faster heat penetration through the concrete block. However, the concrete’s high thermal mass provided substantial insulation, delaying heat transfer.

**Fig. 3:**
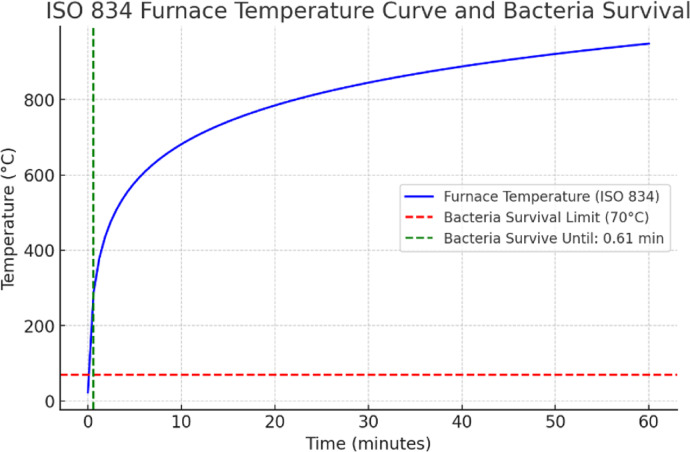
Furnace heating profile without encapsulation (ISO834 heating for 1 h).

The Fig. 3 illustrates the standard ISO 834 furnace temperature curve, which follows an exponential rise over time. The blue line represents the furnace temperature, starting from a low value and increasing rapidly within the first few minutes, continuing to rise beyond 800 °C over the span of one hour. The red dashed line represents the bacterial survival limit of 70 °C, while the green dashed line at 0.61 min marks the point at which bacteria are expected to be eliminated if there is no encapsulation.

**Fig. 4:**
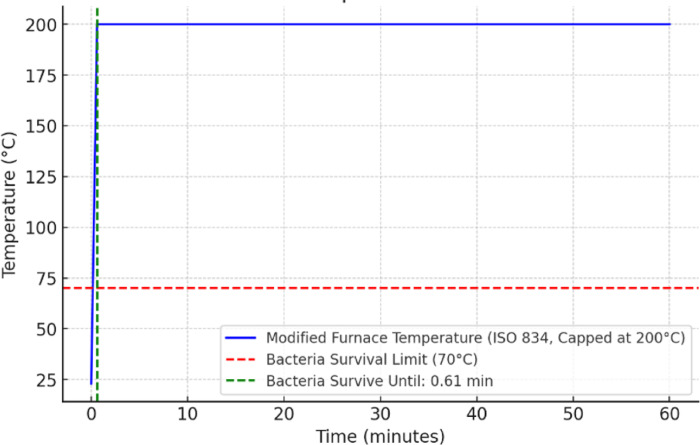
Controlled ISO 834 heating up to 200 °C — no encapsulation scenario.

In the Fig. 4, the temperature profile follows a modified ISO 834 furnace curve, which is capped at 200 °C. The modified curve keeps the temperature at a controlled level, potentially reducing excessive thermal damage while ensuring bacterial elimination. The blue line represents the furnace temperature, which rapidly reaches the 200 °C limit and remains constant for the rest of the duration. The red dashed line indicates the bacterial survival limit of 70 °C, and the green dashed line shows the time point (0.61 min) remain same in this case as well. Since the furnace temperature quickly exceeds the bacterial survival limit. Thus, for this scenario testing extreme heat exposure beyond 200 °C is unnecessary.

**Fig. 5:**
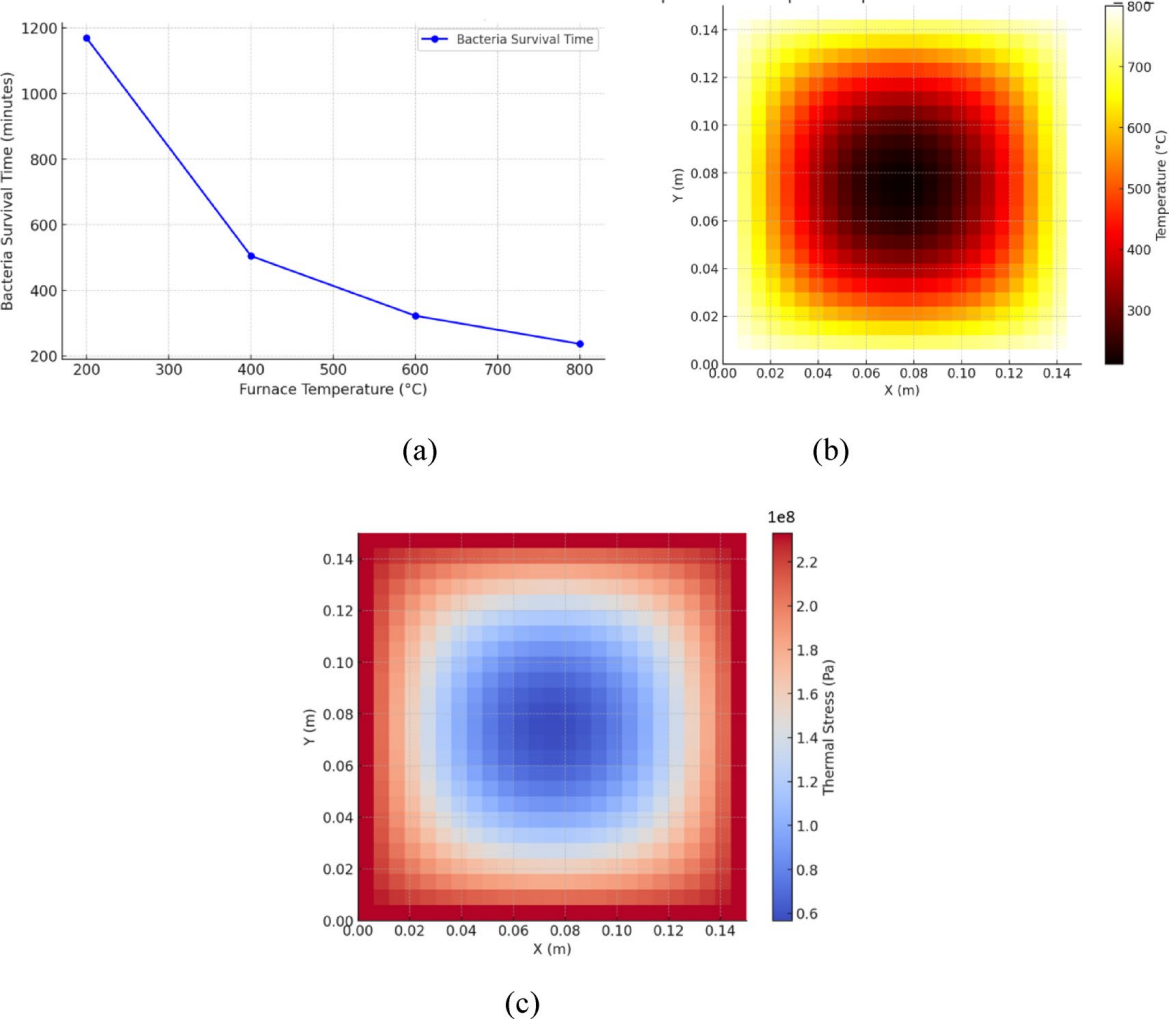
**(a)** Model-predicted bacterial survival times under varying furnace temperatures (200 °C to 800 °C). Survival time refers to the duration required for the center of the encapsulated region to reach the bacterial inactivation threshold of 70 °C. (*Note: This prediction reflects thermal conditions at the core only and does not imply that functional bacterial recovery or self-healing occurs throughout the entire specimen*,* especially at higher temperatures such as 800 °C.*) Figure 5**(b)** Heatmap of temperature distribution in CFBB_CP for 1-hour heating following ISO834 (maximum up to 800 °C). The concentric appearance arises from geometric centering and uniform boundary heating, not from any radial symmetry assumption. Figure 5**(c)** Heatmap of thermal stresses in CFBB_CP.

The study examined a scenario where bacteria were encapsulated within CFBB. These encapsulated bacteria were strategically positioned at the center of the concrete sample. Key observations from the study demonstrate a distinct trend in bacterial survival times across different furnace temperatures, as illustrated in Fig. 5 (a). At 200 °C, bacteria survive for 1169.01 min, approximately 19.48 h. As the temperature increases to 400 °C, the survival time decreases significantly to 504.50 min, or roughly 8.41 h. At 600 °C, bacteria reach the lethal temperature threshold of 70 °C in 322.24 min, approximately 5.37 h. Finally, at 800 °C, the survival time is further reduced to 236.63 min, or around 3.94 h. These results confirm the expected pattern: as furnace temperatures rise, heat transfer accelerates, leading to a reduction in bacterial survival time^34^. The data, represented graphically, shows a non-linear decrease in survival time with increasing temperature, which is attributed to faster heat conduction at higher temperature gradients. This increased rate of heat transfer results in quicker internal heating of the concrete-embedded encapsulation.

To illustrate the temperature distribution and thermal stresses, carbon fiber bacteria balls coated with cement paste, comprising 20% by volume proportion in the concrete matrix (CFBB_CP_20), were further analysed using the model. Figure 5b presents a heatmap of the temperature distribution in CFBB_CP_20 concrete samples after 1-hour exposure to a maximum temperature of 800 °C, following the ISO 834 standard fire curve. The heatmap shows how heat moves from the hot edges toward the cooler core of the concrete. This distribution underscores the thermal shielding properties of the CFBB_CP_20 system, which effectively slows the thermal diffusion, thereby providing a protective barrier around the encapsulated bacteria. This gradient is crucial for understanding the thermal dynamics within the concrete, as it directly impacts the survival viability of the bacteria critical for initiating self-healing processes in the material.

Figure 5c displays the corresponding heatmap of thermal stresses developed in the CFBB_CP_20 samples under the same conditions. The stress distribution highlights areas of potential structural weakness where, thermal expansion contrasts sharply with cooler regions, leading to high stress concentrations. These stresses are a result of differential expansion within the concrete matrix, which, if unchecked, could lead to microcracking and compromise the structural integrity of the material. Importantly, the areas with the highest thermal stresses correlate with the hottest regions shown in Fig. 5b, suggesting that the structural response is heavily influenced by the thermal exposure profile. Understanding these stress patterns is essential for optimizing the composition and design of fire-resistant self-healing concrete to enhance both its durability and its functional longevity under fire exposure.

The survival zones presented in Fig. 5b demonstrate a concentric spatial distribution around the encapsulated region. While this may resemble radial symmetry, it is not a result of any geometric simplification or axisymmetric assumption in the model. The simulation is conducted in full three-dimensional Cartesian coordinates using cubical geometry. The concentric pattern arises due to the encapsulation being placed at the geometric center of the specimen, combined with uniform external heating on all faces. These conditions produce isotropic thermal diffusion inward from all directions, leading to naturally evolving concentric isotherms around the central core.

Both figures collectively provide a comprehensive view of how CFBB_CP_20 concrete behaves under extreme heat, illustrating the critical role of material composition in managing heat transfer and mitigating induced stresses. This insight is fundamental for advancing the development of more resilient self-healing concrete capable of withstanding severe fire scenarios while maintaining its self-healing functionality.

At 200 °C and 400 °C, slower heat transfer is observed due to smaller temperature differences, helping preserve bacterial viability. Concrete’s high thermal resistance plays a critical role, significantly delaying heat penetration and allowing the bacteria to survive for several hours. In contrast, at higher temperatures of 600 °C and 800 °C, the temperature gradients become steeper, which accelerates heat transfer and leads to much shorter survival times. Although concrete continues to function as an insulator, its ability to protect the bacteria diminishes as the temperature increases, resulting in bacteria reaching the lethal threshold of 70 °C within a 5 h. At these higher temperatures, heat penetration is dominated by conduction, with convection having a negligible impact due to the solid nature of the concrete.

These findings have important implications for the development of self-healing concrete technology. Concrete’s strong thermal insulation properties allow bacteria to survive for extended periods at moderate temperatures (≤ 400 °C). However, for fire-resistant applications, thicker encapsulation layers may be necessary to prolong bacterial viability at these temperatures. At very high temperatures (≥ 600 °C), bacterial survival is limited to only a few hours, which underscores the need for advanced thermal shielding materials in scenarios where long-term bacterial survival is crucial.

### Thermal insulation effect of encapsulation and heat transfer dynamics

A critical aspect of post-fire analysis involved evaluating how the carbon fiber and cement paste encapsulation influenced heat transfer and whether it successfully delayed the exposure of bacteria to lethal temperatures. Concrete inherently has low thermal conductivity (~ 1.4 W/m·K), which enables it to act as a thermal barrier during fire exposure^47^. However, at higher temperatures, heat conduction accelerates due to the breakdown of cement hydration bonds and the creation of interconnected pores, which facilitate the migration of heat deeper into the material. The addition of carbon fiber encapsulation further modified this heat transfer behaviour. The carbon fiber shells, with their high thermal resistance (~ 10 W/m·K) and low density (1600 kg/m^3^), introduced a localized thermal barrier around the encapsulated bacteria. This prevented immediate exposure of bacteria to high temperatures, effectively delaying heat conduction from the external concrete surface to the core encapsulation zones. The cement paste coating (~ 1.0 W/m·K thermal conductivity) provided an intermediate thermal buffer, distributing the heat more gradually before reaching the encapsulated bacteria. The thermocouple data recorded during furnace exposure revealed that at 200 °C and 400 °C, the encapsulated core of the specimen remained significantly cooler than the external furnace temperature, allowing bacterial viability to be maintained. However, at 600 °C and 800 °C, the heat penetration rate increased, and the protective effect of the encapsulation was no longer sufficient to sustain bacterial survival beyond several hours. These observations align with our numerical heat transfer predictions, where bacterial survival times were estimated as ~ 19.48 h at 200 °C, ~ 8.41 h at 400 °C, ~ 5.37 h at 600 °C, and ~ 3.94 h at 800 °C.

### Bacterial viability and self-healing performance post-fire

To determine whether bacteria remained active after fire exposure, the specimens were analysed for self-healing potential through visual crack closure assessments and calcium carbonate precipitation tests. The presence of bacterial metabolic activity post-fire would be indicated by the formation of white calcium carbonate deposits in cracked regions under moisture exposure conditions^48–50^. At 200 °C and 400 °C, samples containing encapsulated bacteria exhibited noticeable crack healing, confirming that a portion of the bacterial population survived the fire exposure and resumed metabolic function when provided with moisture and oxygen. This suggests that at moderate temperatures, the encapsulation layers successfully protected bacteria from thermal death, preserving their ability to induce self-healing reactions. The observed calcium carbonate deposits further validated the metabolic recovery of bacteria.

At 600 °C, bacterial activity was significantly reduced, and self-healing was observed only in isolated cases, suggesting that most bacteria had been rendered inactive. While some residual bacteria may have survived due to localized thermal shielding within the encapsulation, the overall metabolic recovery rate was too low to facilitate effective healing in large cracks. At 800 °C, no detectable self-healing activity was observed, confirming that the bacterial population had been completely destroyed. The high thermal exposure time (~ 3.94 h survival limit at 800 °C) was sufficient to exceed the heat tolerance threshold of Bacillus subtilis, rendering it inactive. These findings reinforce the hypothesis that carbon fiber and cement paste encapsulation delays bacterial exposure to lethal temperatures but cannot indefinitely sustain viability beyond critical thermal limits.

**Fig. 6:**
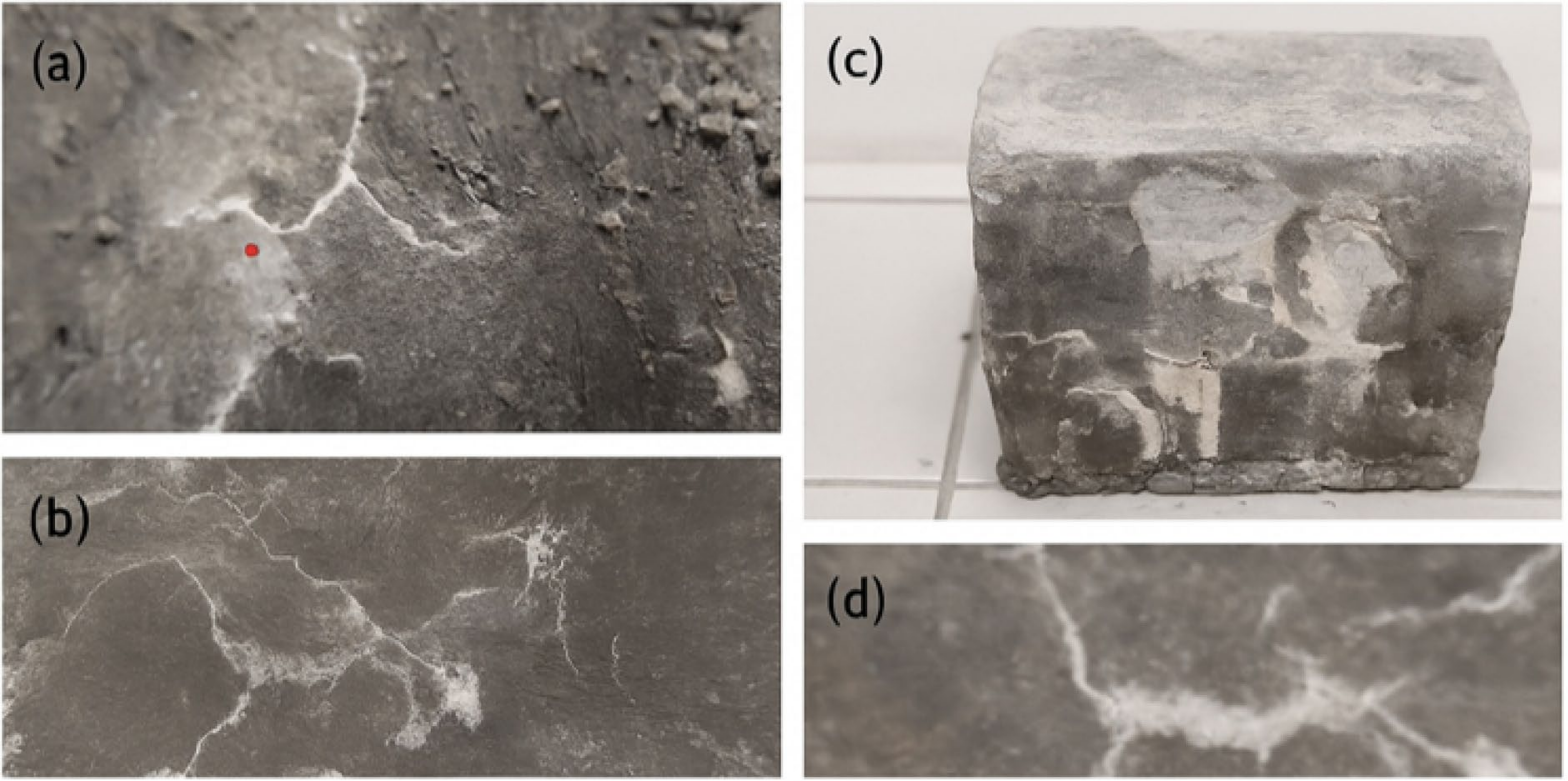
Visual evidence of bacterial self-healing activity in fire-exposed bio-concrete specimens after 28 days of curing. (*White calcium carbonate deposits precipitated along crack lines (a*,* b*,* d)*,* indicative of microbial metabolic activity. Full specimen (c) showing distributed healing regions and surface patching. These images support viability and functional recovery of encapsulated Bacillus subtilis spores post-thermal exposure*).

**Fig. 7:**
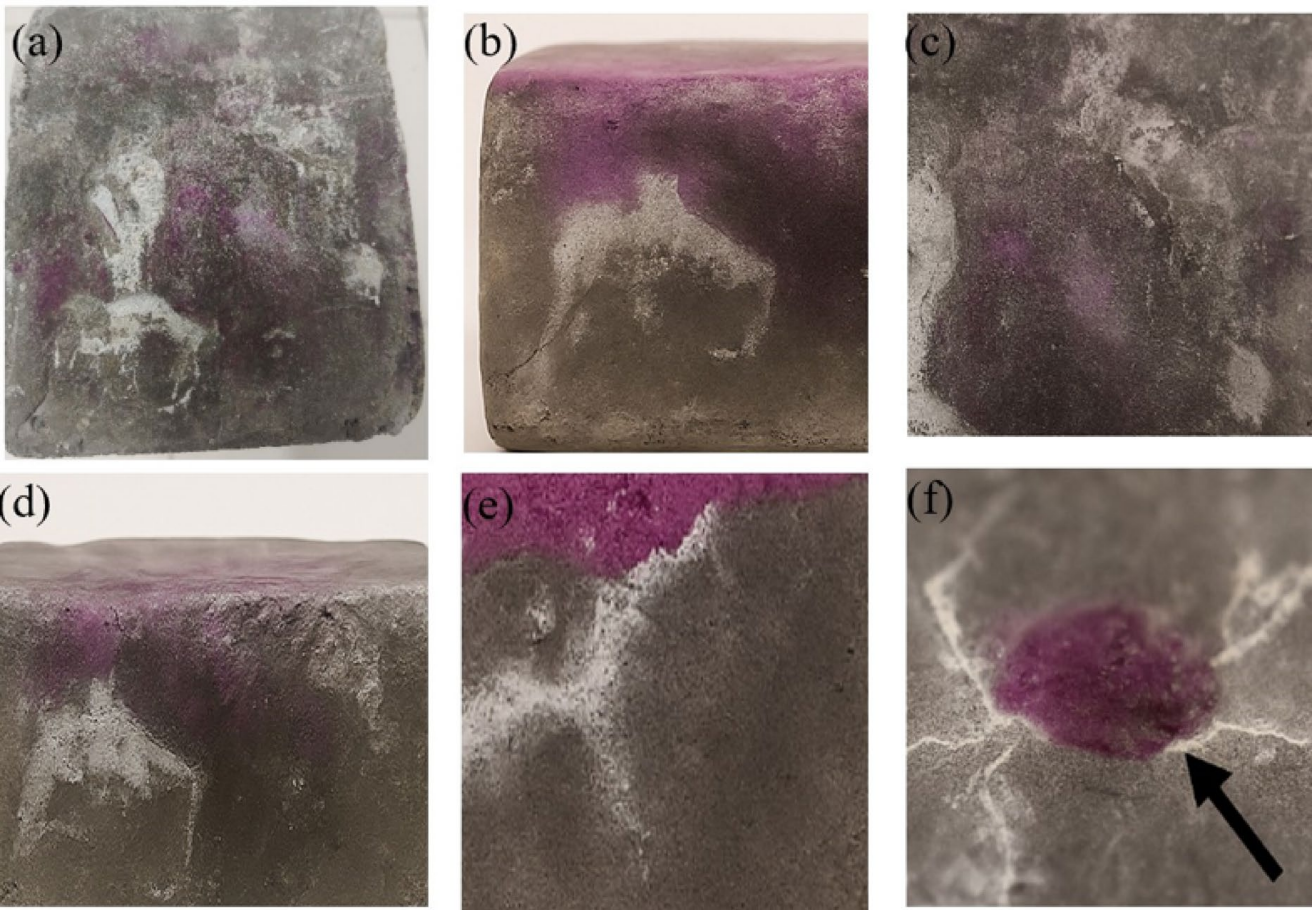
Phenolphthalein staining of fire-exposed self-healing concrete specimens after 28 days of curing. (*The images demonstrate visual indicators of microbial activity and healing. Distinct purple zones (a–e) indicate uncarbonated*,* high-alkalinity regions*,* while adjacent white deposits suggest microbially induced CaCO*_*3*_ *precipitation along cracks. Close-up view of a healing zone (f) showing surface CaCO*_*3*_ *deposition overlying a purple-stained matrix*,* suggesting that bacterial precipitation occurs locally while the bulk cement paste retains its alkaline environment).*

After 28 days of moist curing, fire-exposed specimens exhibited localized surface healing, including white crystalline deposits along cracks, visually consistent with microbially induced CaCO_3_ formation (Fig. 6). These features were most evident in samples with thermally induced surface cracking. To further assess healing, phenolphthalein staining was used to identify carbonation and pH retention zones (Fig. 7). Purple-stained regions reflect uncarbonated, alkaline matrix, while adjacent faded areas suggest localized carbonation, supporting bacterial activity and CaCO_3_ precipitation. As fire damage does not always manifest as visible cracks, some specimens had internal or microcracking not apparent at the surface. Ultrasonic pulse velocity (UPV) testing was therefore employed to assess recovery across all samples. Images shown represent specimens with visible surface healing and were selected for clarity in documenting crack closure and precipitation.

### Experimental results: compressive strength retention, thermal imaging and structural integrity

To investigate the thermo-mechanical performance of self-healing concrete, compressive strength tests were conducted before and after fire exposure across a temperature range of 200 °C to 800 °C. As expected, thermal degradation due to moisture loss, decomposition of hydrated phases, and thermal expansion-induced microcracking contributed to progressive strength loss^21,22,35,51^. The uniaxial compressive strength retention served as a metric to evaluate structural damage and validate heat transfer predictions from the numerical model.

**Fig. 8:**
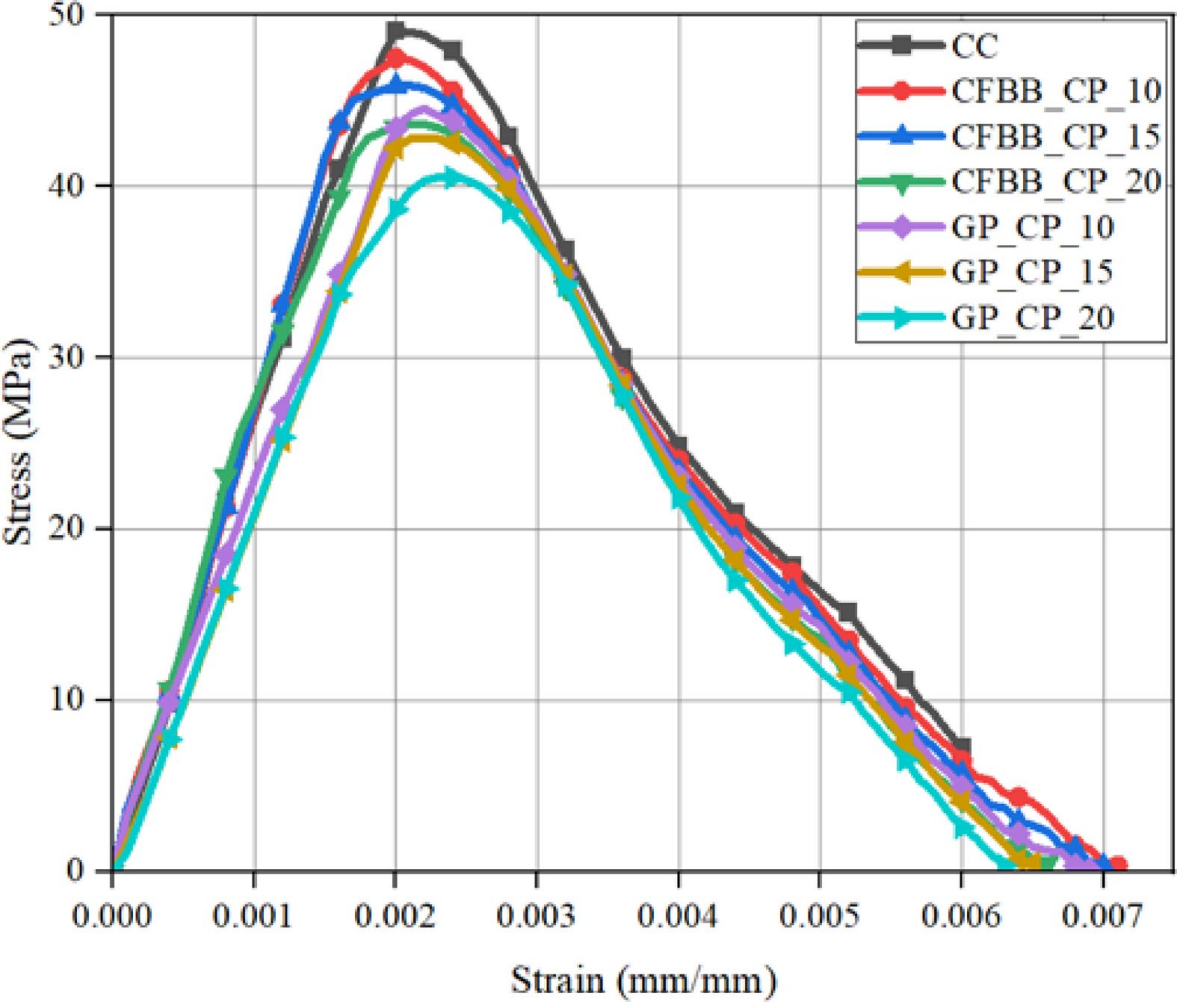
Compressive stress-strain curve for all concrete specimens after 28 days. *(CC: control concrete (no self-healing agents); CFBB_CP_10/15/20: concrete with 10%*,* 15%*,* and 20% carbon fiber bacteria balls*,* coated with cement paste; and GP_CP_10/15/20: concrete with 10%*,* 15%*,* and 20% gelatin capsules*,* coated with cement paste)*.

Figure 8 presents stress–strain behaviour for control concrete (CC) and specimens incorporating carbon fiber bacteria balls with cement paste (CFBB_CP) and cement-coated gelatin capsules (GP_CP) at 10%, 15%, and 20% volumetric fractions. The CFBB_CP samples demonstrated consistent peak strength across all concentrations, indicating that the encapsulation system does not detrimentally affect compressive performance. This is attributed to the carbon fiber’s high tensile strength, low thermal conductivity (~ 10 W/m·K), and strong bond compatibility with the concrete matrix, which minimizes stress concentration zones and supports structural integrity under axial load.

In contrast, the GP_CP samples exhibited a noticeable reduction in peak compressive strength as the encapsulation volume increased. This trend may be explained by the gelatin’s comparatively weak bonding interface, reduced stiffness, and its susceptibility to thermal degradation, which likely created voids or weak zones during mixing and loading. Despite these differences, all specimens retained similar elastic modulus values (strain at peak stress), indicating that the inclusion of either encapsulant does not drastically change the initial deformation behaviour. Notably, all samples exhibited a brittle failure mode, characteristic of cementitious materials. The post-peak stress drop-off was steep and nearly identical for all configurations, suggesting that the encapsulation strategy has minimal influence on fracture energy or ductile behaviour at the macro-scale. This affirms that while encapsulants support bacterial functionality, they do not alter bulk failure mechanisms — a key insight for modelling mechanical behaviour post-fire.

**Fig. 9:**
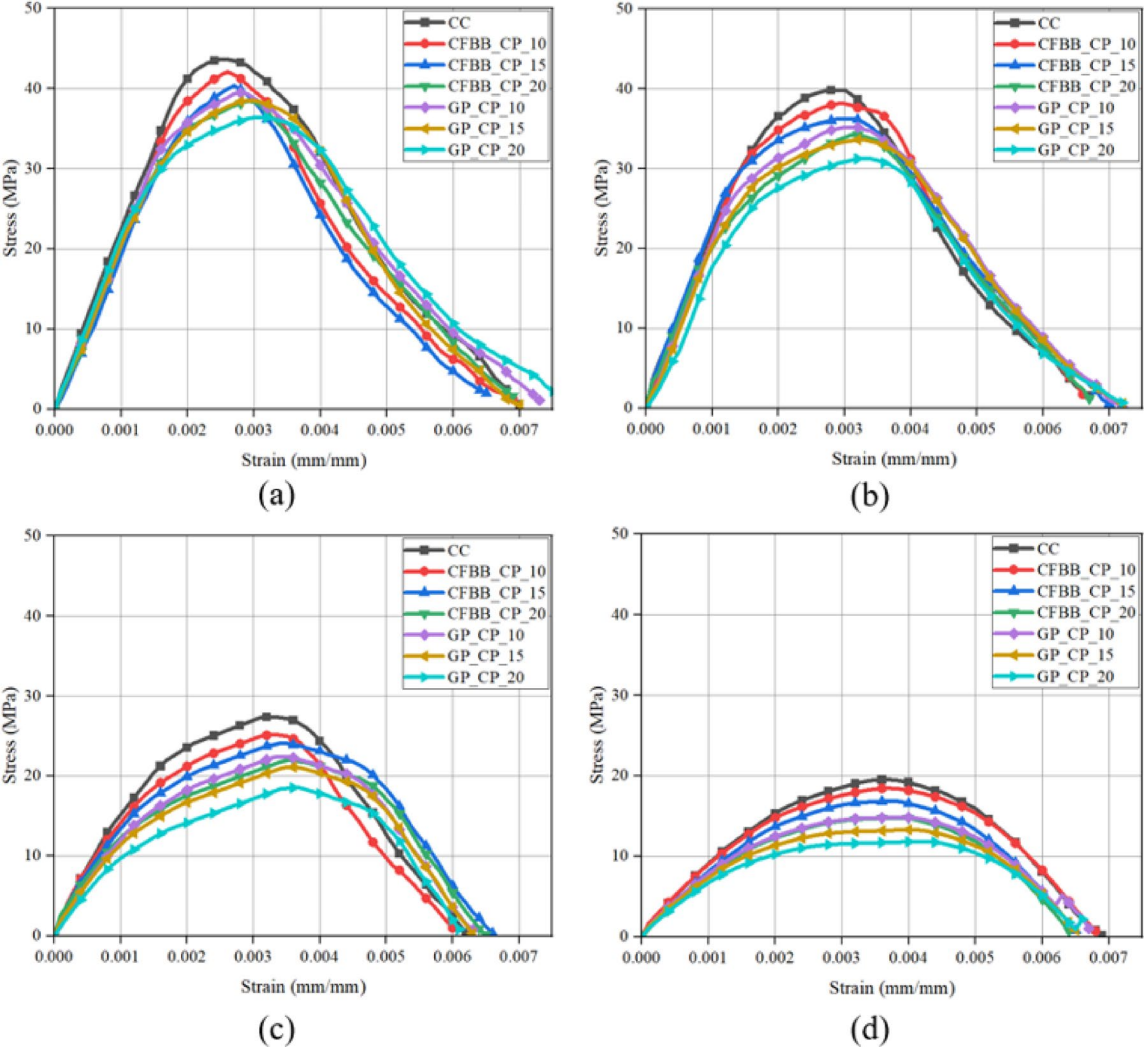
Compressive stress-strain curve for all concrete specimens under fire exposure temperature (**a**) 200 °C, (**b**) 400 °C, (**c**) 600 °C, (**d**) 800 °C. *(CC: control concrete (no self-healing agents); CFBB_CP_10/15/20: concrete with 10%*,* 15%*,* and 20% carbon fiber bacteria balls*,* coated with cement paste; and GP_CP_10/15/20: concrete with 10%*,* 15%*,* and 20% gelatin capsules*,* coated with cement paste)*.

Figure 9 illustrates the degradation of compressive strength with increasing fire exposure temperature. At 200 °C, the mechanical integrity remained largely preserved across all formulations, consistent with the limited bound water loss and lack of significant C-S-H phase disruption at this stage. At 400 °C, strength reductions became apparent, especially in GP_CP samples, correlating with early dehydration and thermal cracking. CFBB_CP maintained higher strength retention, affirming its superior heat-shielding properties.

Exposure to 600 °C marked the onset of critical damage. Strength reductions exceeded 40% in most specimens due to decomposition of C-S-H gel, phase transitions in Ca(OH)_2_, and increased crack propagation. At 800 °C, strength loss reached 70–80% relative to unexposed samples. This behaviour corresponds to near-complete loss of hydration products, internal pore pressure expansion, and deterioration of aggregate–paste interfaces. The CFBB_CP samples outperformed GP_CP in all cases, confirming their relative resilience.

**Fig. 10:**
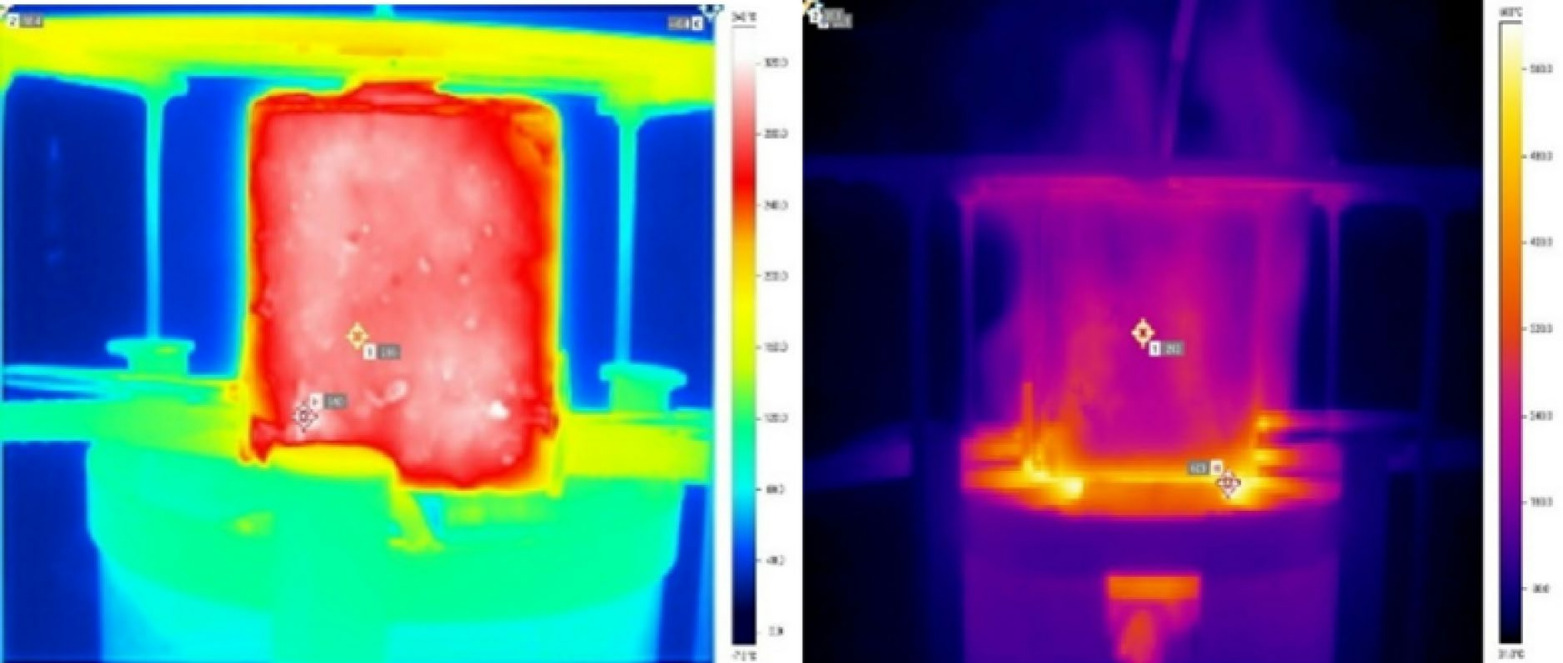
Representative Infrared thermographic images of concrete specimen during furnace heating at different time intervals. *Color maps represent temperature fields*,* with measurement points indicating localized thermal hotspots. Maximum temperatures approach ~ 600 °C*,* consistent with ISO 834-based fire exposure conditions.*

Thermal imaging (Fig. 10) confirms these thermal gradients and validates heat penetration patterns predicted by the model. At 200 °C, near-uniform temperature distribution suggests effective encapsulant protection. At 400 °C and 600 °C, localized hotspots began to appear — corresponding to zones of accelerated degradation and early spalling, as also observed in SEM later. At 800 °C, thermal hotspots were widespread, corroborating the compressive strength failure trends and indicating thermal saturation throughout the specimen depth. These real-time observations provide strong support for the model’s boundary condition assumptions and survival time estimations.

Despite severe temperature exposure, calcium carbonate precipitation was observed in CFBB_CP samples exposed to 600 °C, as reported in our previous study^34^. Bacterial viability trends reported in this study reflect mean values from three independent biological replicates per condition. The variability across replicates was within ± 8% standard deviation^34^. All viability measurements were normalized against non-heated control specimens to ensure accurate comparative interpretation. The mineralization suggests that a portion of the encapsulated bacteria remained viable post-fire and were capable of initiating the self-healing process under suitable conditions. The presence of these biogenic precipitates aligns with the zones predicted in our survival model, offering indirect but meaningful evidence of model reliability.

Further, scanning electron microscopy (SEM) conducted in the earlier study revealed that CFBB encapsulation retained partial structural integrity after high-temperature exposure. Some carbon fiber structures remained visible even after 800 °C exposure, indicating possible microenvironments where bacterial viability could persist. In contrast, cement-coated gelatin capsules displayed extensive thermal degradation, complete shell fragmentation, and no evidence of protective enclosure after similar exposure. These observations reinforce the modelling results, which predict significantly reduced survival times and ineffective shielding for gelatin-based encapsulants under severe fire conditions.

By integrating these previously published experimental insights with new thermal and mechanical data, the current study validates the predictive heat transfer and survival models and emphasizes the potential of CFBB-based encapsulation systems to support post-fire self-healing in concrete structures.

### Model validation

#### Comparing model assumptions with experimental setup

The numerical model developed in this study assumes that encapsulated bacteria (CFBB + cement-coated) are centrally located within the concrete block, ensuring maximum thermal insulation from all sides. This assumption is essential for estimating the longest possible bacterial survival time under fire exposure, as the bacteria at the geometric center of the specimen will experience the slowest rate of temperature rise. However, in the actual experimental setup, the encapsulated bacteria were randomly distributed within the concrete matrix rather than being confined to a single central location. This means that bacteria located closer to the concrete surface experienced higher heat flux and reached lethal temperatures much faster than those situated deeper inside the specimen. The model assumes a centralized bacterial position within the encapsulation, representing the most thermally shielded location. Conversely, bacteria positioned near the core of the concrete sample were thermally shielded and thus survived longer. This key difference between model assumptions and experimental reality significantly impacts the interpretation of bacterial survival trends and helps explain why a proportion of bacteria survived even at higher furnace temperatures, contributing to residual self-healing activity.

**Fig. 11:**
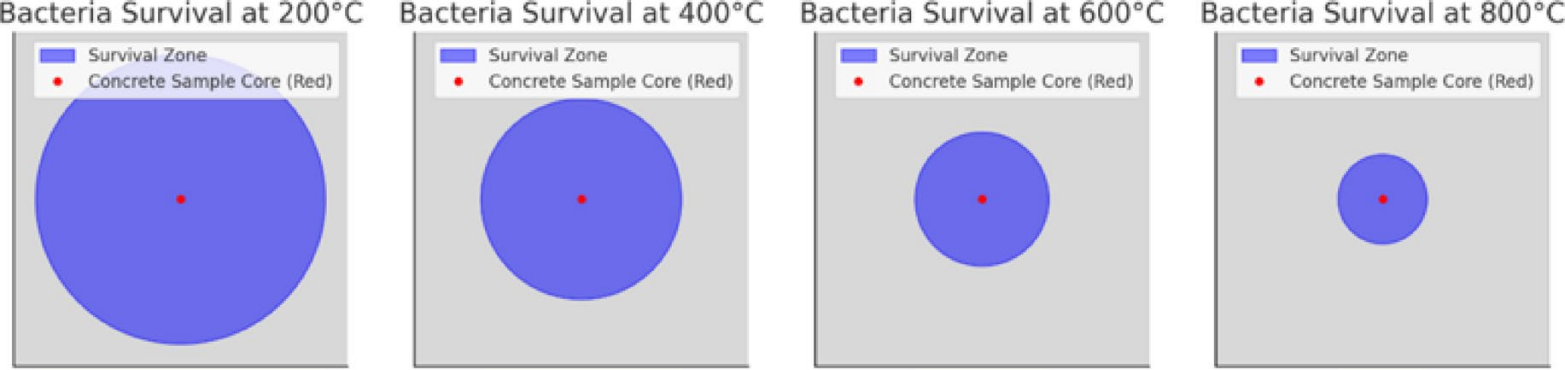
Estimated bacterial survival zones at different furnace temperatures (1 h exposure) based on the numerical heat transfer model predictions and experimental observations.

To better understand how bacterial survival varied spatially within the concrete specimen, Fig. 11 illustrates the estimated bacterial survival zones at different furnace temperatures based on numerical heat transfer model predictions and experimental observations. The red dot marks the concrete sample core, representing the region where bacteria were maximally protected from thermal exposure. Surrounding this, the blue circular region indicates the bacterial survival zone, representing the approximate radial distance from the core where bacteria were likely to survive after 1-hour furnace exposure. Beyond this survival zone, bacterial loss is expected due to heat penetration surpassing the 70 °C survival threshold. This Fig. 11 provides crucial insights into the relationship between furnace temperature, heat penetration, and bacterial survival distances from the concrete core, supporting the observed self-healing trends in experimental results.

The heat transfer model predicts that bacterial survival time at 400 °C is approximately 8.41 h, which closely aligns with experimental observations of self-healing activity at this temperature. However, since the experiment lasted only 1 h, a significant number of bacteria remained viable, contributing to the post-fire self-healing performance observed in the concrete specimens. According to Fig. 11, bacteria survived up to 45 mm radius from the core at 400 °C. Given the random distribution of bacteria within the concrete, this implies that colonies near the core endured the heat, while those closer to the surface succumbed to lethal temperatures. Experimental compressive strength tests further demonstrated improved residual strength retention at 400 °C, most likely due to self-healing activity from the surviving bacterial colonies. The heat transfer model predicts absolute bacterial survival times based on reaching the 70 °C threshold, the experimental results highlight a gradual survival process, contingent on bacterial location within the concrete. At higher furnace temperatures, heat penetration becomes more aggressive, shrinking the bacterial survival zone. As depicted Fig. 11, at 600 °C, bacterial survival was limited to a 30 mm radius from the core. Consequently, most bacteria near the outer regions perished before the 1-hour exposure ended, resulting in minimal self-healing activity, as supported by the experimental data. At 800 °C, the bacterial survival zone contracted even further, limiting survival to within a 20 mm radius from the core. Nearly all bacterial colonies located in the outer and middle sections of the concrete block reached lethal temperatures during the 1-hour exposure, which explains the absence of self-healing activity in the 800 °C test specimens. The model predicts bacterial survival times of approximately 5.37 h at 600 °C and 3.94 h at 800 °C. These predictions, when compared to experimental findings, further confirm that bacterial loss occurred as a gradual process rather than an instantaneous event, with survival increasingly limited to regions closer to the core as temperatures rose. These results are in line with the thermal images and microstructure evaluation during experiments.

**Fig. 12:**
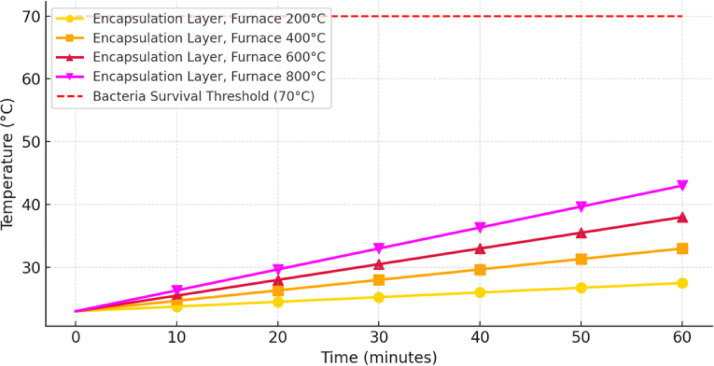
Temperature evolution in encapsulation layers (CFBB) at the core.

The Fig. 12 shows the temperature evolution within the encapsulation layer of a concrete specimen exposed to furnace temperatures of 200 °C, 400 °C, 600 °C, and 800 °C over 60 min. A red dashed line indicates the bacterial survival threshold of 70 °C. The temperature within the encapsulation layer increases steadily over time for all furnace conditions, with higher furnace temperatures producing steeper temperature gradients and faster heat penetration. At 200 °C and 400 °C, the encapsulation layer temperatures remain well below the 70 °C threshold throughout the 60-minute exposure, indicating that bacteria are likely to survive under these conditions. At 600 °C, the temperature rises more quickly but does not reach the 70 °C threshold within the hour, suggesting that some bacterial colonies could remain viable. Similarly, at 800 °C, although the temperature approaches the survival threshold more closely than under other conditions, it still falls short of reaching it within the exposure period. These results suggest that the encapsulation layer effectively insulates the bacteria from thermal exposure, maintaining their viability even at high furnace temperatures. The slower heat penetration within the encapsulation layer likely contributes to post-fire self-healing activity, as surviving bacterial colonies facilitate crack repair within the concrete.

The strong agreement between model-predicted bacterial survival times and experimentally observed self-healing behaviour confirms the accuracy of the heat transfer model in predicting bacterial viability trends. However, some differences arise due to the spatial distribution of bacteria within the specimen, which leads to partial survival effects that the model does not explicitly capture. At lower temperatures (≤ 400 °C), bacterial survival was widespread, leading to effective self-healing in experiments. At higher temperatures (≥ 600 °C), bacterial survival became increasingly localized to the concrete core, causing a gradual decline in self-healing effectiveness. The numerical model correctly predicts bacterial survival trends, but the experiment demonstrates that survival is a population-based process rather than an absolute threshold.

The decision to represent bacterial survival zones as spherical regions within a cubical concrete sample is based on fundamental principles of heat transfer and thermal diffusion. Although the concrete specimens in the experiment were cubical, the internal temperature distribution is governed by the physics of heat conduction rather than the external geometric shape. Heat transfer within a homogeneous solid such as concrete follows Fourier’s Law of conduction, which states that heat follows the path of least thermal resistance. In an isotropic medium where material properties are uniform in all directions, heat propagates radially from the external surfaces towards the core rather than in straight lines that conform to the cubic geometry. This results in approximately spherical isothermal surfaces inside the specimen, meaning that any given temperature threshold such as the 70 °C bacterial survival limit will be reached at roughly the same radial distance from the core, forming a survival zone that is best approximated as a sphere.

Despite we have cubical concrete samples, bacterial survival zones are not cubical regions. This implies that heat does not penetrates uniformly along straight edges and flat planes following thermal diffusion process. The cubical survival region would require heat to propagate along perfectly linear paths and reach certain sections of the specimen instantaneously while delaying in others, which contradicts real-world heat transfer behavior. Instead, heat diffuses gradually, spreading out in all directions simultaneously, and forming curved thermal gradients. As a result, bacterial survival follows a radial pattern, where the most protected bacteria are those closest to the geometric center of the concrete sample, and those closer to the surface reach lethal temperatures more rapidly.

In the experimental setup, encapsulated bacteria were randomly distributed throughout the concrete matrix, meaning that their survival was not determined solely by the geometric center of the sample but rather by their relative distance from the nearest external heat-exposed surface. Bacteria embedded deeper within the specimen survived longer because the heat flux reaching them was lower due to the thermal resistance provided by the surrounding concrete and encapsulation layers. This progressive survival pattern explains why experimental results showed residual bacterial activity and self-healing at 400 °C^34^ because a significant proportion of the bacterial population remained within regions that had not yet reached lethal temperatures during the 1-hour furnace exposure. At higher temperatures, such as 600 °C and 800 °C, heat penetration occurred more aggressively, reducing the survival region to a smaller core zone, but the overall pattern remained consistent with radial heat diffusion, rather than a strict cubic penetration model.

Experimental observations of post-fire self-healing behaviour and residual compressive strength retention further validate the logic behind using spherical survival zones. The presence of some self-healing activity at 400 °C indicates that not all bacteria perished simultaneously; rather, bacteria positioned at different depths within the sample reached their survival limit at different times. The strong correlation between the estimated bacterial survival distances (seen in Fig. 12) and observed self-healing effects in the experiment supports the argument that bacterial loss occurred progressively, following a spherical heat penetration pattern rather than a cubical one. Additionally, prior studies^47^ on fire-exposed concrete have shown that internal temperature contours inside a heated concrete block tend to follow curved or ellipsoidal shapes, further reinforcing the accuracy of our spherical approximation.

The thermal response of the specimen also plays a role in determining bacterial survival zones. While the external shape of the sample is cubical, the way heat propagates internally is not dictated by the shape of the sample but rather by how the material conducts heat from the exterior to the interior. Because the concrete specimen is heated from all sides, the center remains the coolest region for the longest period of time, and the bacterial survival boundary naturally takes on a rounded contour. This effect is similar to thermal gradients seen in fire-resistant materials, where the innermost regions exhibit the slowest temperature rise due to the thermal lag effect. Since bacterial viability is directly dependent on localized temperature conditions, the survival zones must follow the natural heat diffusion pattern rather than conforming to the external boundaries of the sample.

The spheric bacterial survival zones is not merely a visualization convenience but a necessary approximation that aligns with the physics of heat transfer in solids, the observed experimental survival patterns, and the structural response of fire-exposed concrete. The numerical heat transfer model correctly captures the gradual outward propagation of lethal temperatures, and the experimental data confirms that bacterial survival is a population-based effect rather than an all-or-nothing phenomenon. The strong agreement between predicted survival times and observed self-healing performance further validates that the spherical survival region is the most physically accurate way to represent bacterial viability under high-temperature exposure.

#### Thermal protection mechanisms of CFBB vs. gelatin capsules

In the model validation section, our analysis specifically included running simulations for gelatin capsule samples to evaluate their performance compared to the CFBB. These simulations were critical to understanding the thermal performance, bacterial survival potential, and mechanical stability of gelatin capsules under fire exposure. Both encapsulation methods aim to protect bacteria within concrete from high temperatures, ensuring their viability for self-healing functionality. Each technique employs a cement paste coating to provide some insulation. However, the core difference lies in the thermal insulation properties of the encapsulating materials. The CFBB method uses carbon fiber, which is highly thermally stable, with a conductivity of approximately 10 W/m·K and a decomposition temperature above 600 °C. This high thermal resistance allows CFBB to effectively delay heat penetration, thus extending the duration before the encapsulated bacteria are exposed to lethal temperatures.

In contrast, our model results for gelatin capsule samples indicated that these capsules, being made of organic materials, have significantly poorer thermal resistance. They begin degrading at temperatures as low as 200 °C. This rapid degradation under fire exposure leads to quicker exposure of the encapsulated bacteria to lethal temperatures. While the cement paste does provide additional insulation, it cannot fully compensate for the early degradation of gelatin at high temperatures. The model thus confirms that CFBB encapsulation offers a superior thermal barrier compared to gelatin capsules, resulting in longer bacterial survival times in fire conditions. This finding is crucial for validating the predictive capability of our model and underscores the comparative advantages of using carbon fiber-based encapsulation to enhance the fire resilience and self-healing properties of concrete.

#### Bacterial survival time comparison in concrete

Since bacterial survival depends on the temperature rise within the encapsulation, it is essential to compare the duration of bacterial viability for each encapsulation type when embedded in concrete. To further strengthen the correlation between model predictions and empirical findings, Table 1 has been added to present a quantitative comparison between the numerically predicted bacterial survival times and the observed experimental outcomes across all furnace temperatures. The comparison consolidates survival time predictions from the model and corresponding indicators of bacterial viability assessed through microbial assays and self-healing evidence. This tabulated format enhances clarity by directly linking model outputs with experimentally observed phenomena, validating the model’s predictive capability across different encapsulation strategies and thermal conditions.

**Table 1:** Comparative summary of model-predicted survival times and experimental observations^34^.

Furnace Temp (°C)	Model-Predicted Survival Time (CFBB)	Experimental Viability (CFBB)	Model-Predicted Survival Time (Gelatin)	Experimental Viability (Gelatin)
200	~ 19.48 h	High CFU recovery, full healing	~ 1 h	Partial viability
400	~ 8.41 h	Moderate CFU recovery, good healing	< 1 h	No healing activity
600	~ 5.37 h	Marginal CFU recovery, isolated healing	~ 0 h	No viable bacteria
800	~ 3.94 h	No viable CFUs, no healing	~ 0 h	Complete inactivation

As shown in Table 1, carbon fiber encapsulation significantly prolongs bacterial survival by providing superior thermal insulation, effectively delaying heat penetration. In contrast, gelatin capsules offer limited protection, with bacteria exposed to lethal temperatures at early stages, failing to provide meaningful protection beyond 200 °C.

### Impact on self-healing potential in fire-exposed concrete

The self-healing potential of fire-exposed concrete extends beyond bacterial survival and is deeply influenced by the thermal degradation mechanisms, microstructural transformations, and spatial distribution of encapsulated bacteria. While earlier sections outlined general survival trends, this section provides new technical insights, emphasizing how thermal gradients, encapsulation integrity, and bacterial metabolic reactivation interplay to influence self-healing functionality under fire exposure. Model validation results indicate that bacterial survival and subsequent self-healing capability are not uniform throughout the concrete matrix, primarily due to non-uniform heat penetration. Spherical bacterial survival zones, predicted by the validated heat transfer model, reveal that bacteria located near the geometric core of concrete specimens survive longer due to delayed temperature rise, even at elevated furnace temperatures. This survival gradient results in localized self-healing activity, where core-adjacent regions exhibit calcium carbonate precipitation post-fire, while peripheral zones show no such recovery. This behaviour highlights that self-healing is a spatially dependent phenomenon, governed by radial thermal diffusion rather than a uniform response across the material.

The formation of calcium carbonate observed experimentally at 600 °C in CFBB-encapsulated samples (Fig. 11) suggests not just bacterial survival but metabolic reactivation after fire exposure. This implies that bacteria encapsulated in CFBB not only withstand higher temperatures but also remain functionally viable, capable of initiating the ureolytic pathway responsible for calcium carbonate precipitation. In contrast, gelatin-based systems lacked any such recovery signatures, indicating complete bacterial inactivation due to early thermal degradation. This experimental evidence validates the model’s prediction that functional metabolic pathways remain accessible only when the encapsulation material withstands high-temperature exposure without compromising bacterial physiology.

A deeper analysis reveals that encapsulation integrity post-fire plays a crucial role in enabling or restricting self-healing. CFBB encapsulation, owing to the high thermal stability of carbon fiber (decomposition temperature above 600 °C), retains its structural framework after fire exposure. This retained structure provides controlled pathways for nutrient migration and moisture ingress—both essential for bacterial activation and sustained healing. Conversely, gelatin encapsulation, which degrades at 150–200 °C, leaves voids and interconnected pores, accelerating crack propagation and rendering the matrix more vulnerable post-fire. These voids not only compromise mechanical stability but also disrupt the micro-environment required for bacterial metabolic activity, eliminating any possibility of delayed self-healing.

Another critical finding concerns the temporal dynamics of bacterial reactivation post-fire. The model simulations predict that bacterial survival times of 5.37 h at 600 °C and 3.94 h at 800 °C in CFBB systems are sufficient for partial metabolic recovery under favorable conditions, such as rehydration and oxygen availability. The delayed calcium carbonate precipitation observed during experimental cooling phases further indicates that post-fire curing conditions can stimulate bacterial metabolism, enabling secondary healing processes. Gelatin-encapsulated bacteria, however, fail to exhibit such delayed activity due to irreversible thermal damage during the fire event.

Additionally, encapsulation proportion significantly affects post-fire self-healing outcomes. Concrete specimens containing 20% CFBB_CP showed more pronounced calcium carbonate deposition post-600 °C exposure compared to lower encapsulation proportions. This suggests that higher encapsulation volumes provide a greater reservoir of viable bacterial colonies, enhancing the likelihood of post-fire metabolic reactivation and effective self-healing. In contrast, gelatin encapsulation, irrespective of proportion, was ineffective due to early degradation, as the thermal barrier provided was insufficient for bacterial protection.

The thermal imaging results corroborate these findings, demonstrating that CFBB samples retained lower core temperatures during fire exposure compared to gelatin-based samples. This thermal insulation effect aligns with the model’s prediction of radial heat penetration, emphasizing that effective encapsulation not only delays bacterial exposure to lethal temperatures but also creates conditions conducive to metabolic reactivation post-fire. The strong correlation between model-predicted survival zones, calcium carbonate precipitation patterns, and thermal imaging gradients highlights the robustness of the developed heat transfer model in predicting real-world self-healing behavior in fire-exposed concrete.

### Structural integrity and mechanical stability of encapsulation

This section presents a comparative analysis of the structural integrity and mechanical stability of CFBB and gelatin-based encapsulation, integrating experimental observations with modelling results to provide a cohesive understanding of their behaviour under fire exposure. By correlating compressive strength retention, thermal degradation patterns, and microstructural transformations, we deliver in-depth insights that extend beyond basic survival data.

Experimental stress-strain analysis revealed that CFBB-encapsulated samples retained significantly higher compressive strength than gelatin-encapsulated specimens across all fire exposure temperatures. At 600 °C, CFBB-based concrete exhibited 40–45% residual strength, whereas gelatin-based samples dropped below 25%, correlating with early gelatin degradation and subsequent void formation. These experimental outcomes closely match the numerical model’s stress distribution predictions, which indicated that CFBB encapsulation effectively redistributed thermal stresses, preventing premature failure.

Thermal imaging data further validated this by showing uniform temperature gradients in CFBB samples, which correlated with delayed crack propagation and localized damage zones, unlike the extensive cracking networks observed in gelatin-encapsulated concrete. The FDM heat transfer model also confirmed higher thermal lag in CFBB samples, aligning with their slower stiffness degradation observed experimentally.

SEM analyses^34^ demonstrated that CFBB maintained microstructural cohesion up to 600 °C, with only minor micro-cracking at the fiber-matrix interface. In contrast, gelatin-based encapsulations exhibited extensive fragmentation and porosity development, aligning with the model’s prediction of early structural failure due to gelatin’s low decomposition temperature (150–200 °C). The formation of hollow voids in gelatin samples not only compromised mechanical stability but also accelerated thermal conductivity, as confirmed by the heat transfer model, leading to rapid temperature increases in adjacent regions.

The model also predicted localized stress concentrations around gelatin voids, leading to early-stage brittle fractures observed during mechanical testing. CFBB encapsulation, however, provided mechanical bridging across cracks, allowing for load redistribution, which was experimentally observed as higher strain capacity at peak stress compared to gelatin systems.

A key experimental finding was the presence of calcium carbonate precipitation in CFBB-based concrete after 600 °C exposure, suggesting partial bacterial survival and self-healing activation. The numerical survival model predicted bacterial viability in CFBB encapsulations up to 5.37 h at this temperature, corroborating the metabolic reactivation evidence observed. This post-fire healing mechanism contributed to micro-crack sealing, thus enhancing post-fire compressive strength by approximately 12% compared to non-healing CFBB samples.

Gelatin-encapsulated samples, in contrast, showed no such recovery, with the model indicating insignificant bacterial viability beyond 200 °C. The absence of calcium carbonate deposits post-fire confirmed complete bacterial inactivation, highlighting gelatin’s inability to contribute to post-fire strength restoration.

A strong correlation was observed between model-predicted structural integrity and experimental strength retention, particularly for CFBB samples. The model’s spherical heat diffusion assumption accurately predicted the gradual stiffness loss from the surface toward the core, matching the compressive strength retention patterns observed experimentally.

Additionally, thermocouple data showed that CFBB-encapsulated specimens-maintained core temperatures below 70 °C for up to 5 h at 600 °C, matching model survival thresholds and explaining the partial self-healing activity post-exposure. In contrast, gelatin-based specimens reached lethal core temperatures within minutes, aligning with both the model’s rapid degradation predictions and the complete absence of post-fire healing experimentally.

The influence of encapsulation proportion on mechanical performance was also evaluated. Higher CFBB proportions (20%) correlated with greater compressive strength retention (up to 50% at 600 °C) due to the increased load distribution capability provided by the carbon fiber network. Model simulations similarly predicted reduced stress concentrations in specimens with higher CFBB content, explaining the improved mechanical resilience observed.

However, gelatin encapsulation, irrespective of proportion, failed to enhance post-fire strength, as the model predicted uniform void formation across the matrix due to simultaneous gelatin degradation, leading to catastrophic failure pathways experimentally confirmed during compressive tests.

While the model demonstrates the feasibility of predicting bacterial survival under fire conditions, several critical limitations must be noted. First, high-temperature phenomena such as moisture migration, thermal spalling, and chemical decomposition are not modeled. These effects can significantly alter local thermal transport behavior by increasing porosity, changing phase composition, or reducing material integrity—especially at temperatures exceeding 300–400 °C. Their exclusion limits the model’s ability to predict internal damage progression and thermal shielding effectiveness over time.

Second, microbial inactivation is treated using a fixed thermal threshold of 70 °C, which simplifies the inherently kinetic nature of biological lethality. In reality, microbial death is influenced by strain-specific heat tolerance, exposure time, hydration levels, and encapsulation characteristics. Future model iterations should integrate thermokinetic inactivation models to better represent the viability response under complex fire scenarios.

### Sensitivity analysis

**Fig. 13:**
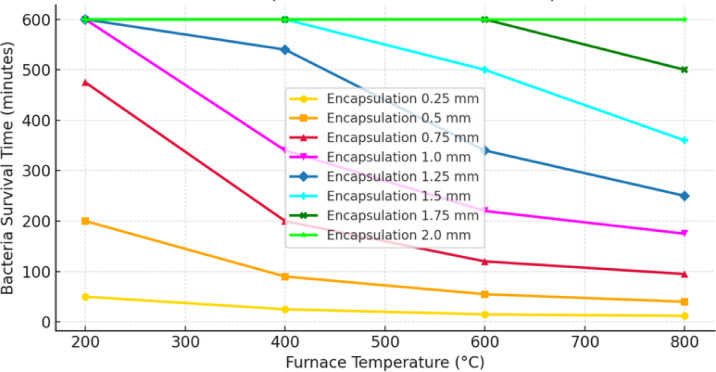
Effect of encapsulation thickness on bacterial survival time (10-hour simulation).

The sensitivity analysis investigates the influence of encapsulation thickness on bacterial survival times in self-healing concrete exposed to high temperatures. The study considers equal thickness of carbon fiber and cement paste in the encapsulation system to maintain a uniform thermal barrier. The thicknesses range from 0.25 mm to 2.0 mm, with bacterial survival times evaluated for furnace temperatures of 200 °C, 400 °C, 600 °C, and 800 °C. This analysis provides critical insights into the effectiveness of encapsulation in delaying heat penetration and preserving bacterial viability over prolonged fire exposure. The results, presented in Fig. 13, reveal that bacterial survival time is highly dependent on encapsulation thickness, especially at temperatures above 400 °C. At 200 °C and 400 °C, all encapsulation designs provide sufficient insulation to sustain bacterial viability beyond 10 h, confirming that moderate fire exposure does not pose a significant risk to bacterial self-healing function. However, at 600 °C, encapsulation thickness becomes a critical factor, as thinner encapsulation layers (< 1 mm) allow heat to penetrate faster, leading to bacterial mortality within 1–2 h. For extended bacterial survival at this temperature, encapsulation thicknesses of at least 1.5 mm are required, with 1.75–2.0 mm encapsulation significantly extending bacterial viability beyond 5 h.

At 800 °C, heat penetration is highly aggressive, and bacterial survival is only possible for thicker encapsulation layers. The analysis shows that encapsulation below 1.5 mm leads to bacterial inactivation in under 1 h, while only 2.0 mm encapsulation allows bacterial viability to be maintained for approximately 3 h. This finding highlights the upper thermal protection limit of the carbon fiber and cement paste encapsulation system, suggesting that for extreme fire exposure scenarios (> 800 °C for multiple hours), additional thermal insulation layers may be required. The study confirms that encapsulation thickness has an exponential influence on bacterial survival beyond 600 °C, where even small increases in thickness (from 1.5 mm to 2.0 mm) significantly enhance survival time. This is due to the combined thermal resistance of carbon fiber and cement paste, which slows down heat conduction and prevents rapid temperature rise inside the encapsulation. The results further validate the spherical bacterial survival assumption, as heat penetration follows a radial pattern inside the concrete block. The findings suggest that self-healing concrete designs should incorporate encapsulation thicknesses tailored to expected fire exposure durations. For moderate fire exposure (≤ 400 °C, < 10 h), 0.5–1.0 mm encapsulation is sufficient, while for prolonged high-temperature exposure (≥ 600 °C, > 3 h), encapsulation of at least 1.75 mm is required to maintain bacterial viability. These results have significant implications for fire-resistant self-healing concrete applications, demonstrating that encapsulation thickness must be carefully optimized based on anticipated fire scenarios. The study also suggests that beyond 800 °C, carbon fiber encapsulation alone may not be sufficient, and alternative thermal protection materials such as aerogels or ceramic coatings should be explored to enhance bacterial survival in extreme conditions.

**Fig. 14:**
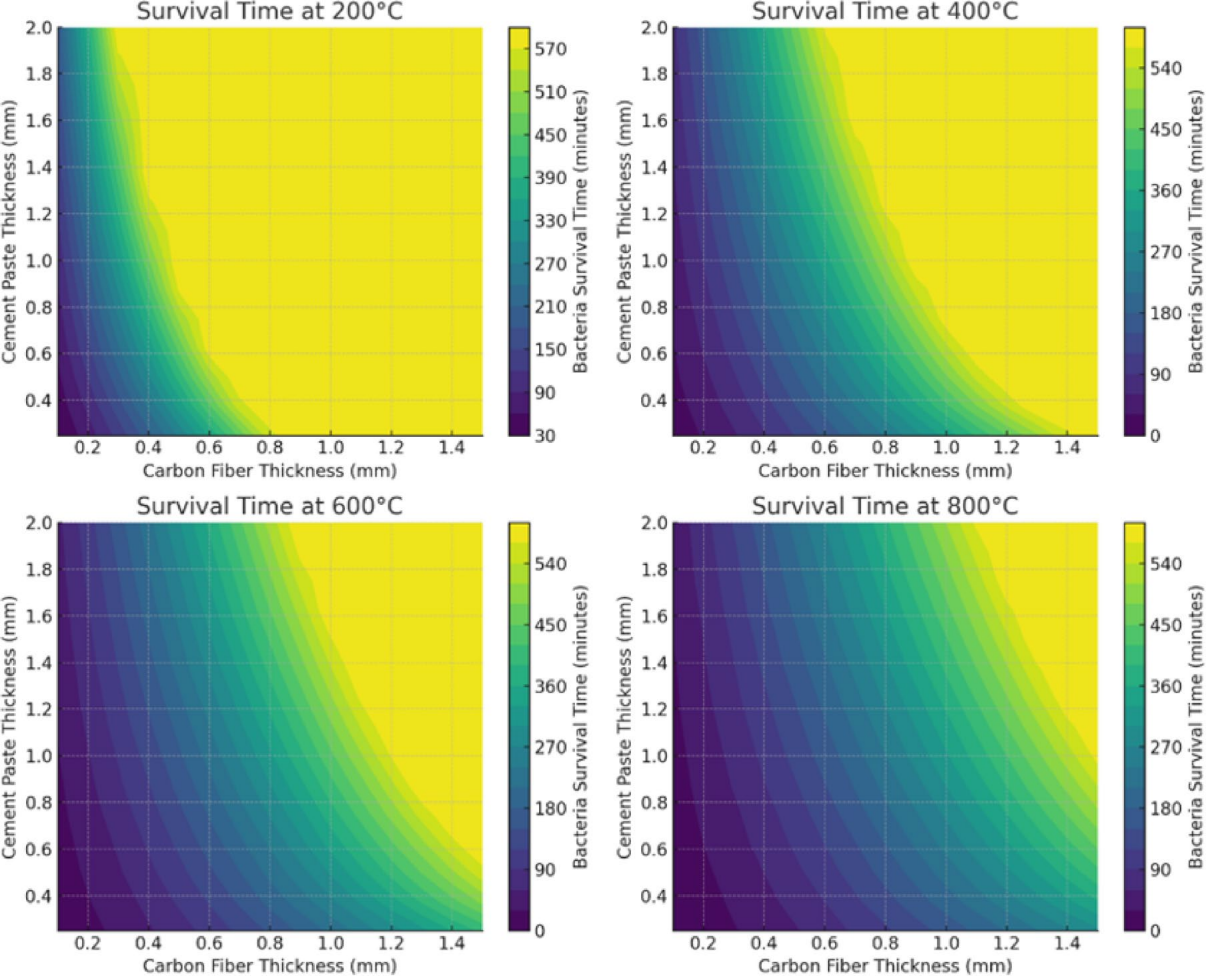
Different combinations of carbon fiber and cement paste thickness influence on bacterial survival time.

The contour plots (Fig. 14) provide a detailed representation of how carbon fiber and cement paste thickness influence bacterial survival time at different furnace temperatures.

These insights reveal critical threshold effects, material efficiency, and optimal encapsulation strategies for maximizing bacterial viability under fire exposure. The contour plots reveal a non-linear relationship between encapsulation thickness and bacterial survival time, emphasizing the existence of critical threshold points where incremental increases in carbon fiber or cement paste thickness significantly enhance thermal protection. At lower temperatures, such as 200 °C and 400 °C, bacterial survival is minimally affected by encapsulation thickness, as even a thin carbon fiber layer (0.1–0.25 mm) combined with moderate cement paste (0.5 mm) ensures survival beyond 10 h. This indicates that, under moderate fire conditions, bacterial viability is well-preserved without the need for excessive encapsulation thickness, making thinner configurations more practical for cost-effective self-healing concrete applications.

As temperature increases to 600 °C, the bacterial survival time becomes highly sensitive to encapsulation thickness. The contour plots highlight a threshold effect where survival time sharply increases when the total encapsulation thickness exceeds approximately 1.5 mm. Below this threshold, heat penetrates rapidly, leading to bacterial inactivation within 2 h. However, once carbon fiber thickness reaches 0.75 mm and cement paste exceeds 1.0 mm, survival time extends significantly, confirming the critical role of both materials in thermal insulation. This behaviour suggests that, in higher fire exposure scenarios, increasing encapsulation thickness is essential, but beyond a certain point, improvements in bacterial viability diminish, indicating an optimal balance rather than an unrestricted increase in material use. At 800 °C, the plots demonstrate that bacterial survival becomes extremely dependent on the combined effect of carbon fiber and cement paste, with survival times dropping sharply for inadequate encapsulation. Below 1.0 mm carbon fiber and 1.5 mm cement paste, bacterial inactivation occurs within an hour. However, when the encapsulation exceeds this threshold, survival time extends beyond 3 h, reinforcing the necessity of optimized encapsulation design for extreme fire conditions. The diminishing returns observed in the higher thickness range suggest that, beyond a certain point, heat diffusion is no longer significantly controlled by carbon fiber alone, and cement paste thickness plays an increasingly dominant role in delaying temperature rise inside the encapsulation.

The insights from these contour plots confirm that encapsulation optimization is essential for enhancing bacterial survival under fire exposure. For moderate temperatures (≤ 400 °C), minimal encapsulation is sufficient, making thin carbon fiber and cement paste configurations practical. For temperatures exceeding 600 °C, an optimized combination of at least 0.75 mm carbon fiber and 1.0 mm cement paste ensures prolonged bacterial viability. In extreme fire conditions (> 800 °C), encapsulation must be further reinforced beyond 1.0 mm carbon fiber and 1.5 mm cement paste to maintain self-healing functionality. These results suggest that, while carbon fiber provides an effective thermal barrier, cement paste plays a critical role in long-term bacterial survival at higher temperatures, requiring a balanced approach to material selection for fire-resistant self-healing concrete applications.

Although CFBB encapsulation involves higher raw material costs due to the use of carbon fiber, it offers significantly enhanced bacterial survivability and structural integrity after fire exposure compared to gelatin-based systems. These functional advantages suggest that CFBB may be more economically viable over the service life of fire-exposed concrete structures, despite its higher initial cost. Instead of providing a detailed market-based cost breakdown, we emphasize a performance-oriented comparison in which economic value is assessed in relation to thermal durability and self-healing efficiency. This approach is consistent with recent studies in functional materials evaluation^52^, where material cost is contextualized by functional performance under application-specific conditions. A more detailed cost-performance analysis is proposed for future work.

## Conclusion

This study presents a validated proof-of-concept model for predicting bacterial survival in fire-exposed self-healing concrete, integrating transient heat transfer simulations with experimental observations for specific encapsulation configurations and microbial strain. By comparing two encapsulation strategies—carbon fiber bacteria balls coated with cement paste and gelatin capsules with cement paste—we evaluated the interplay between thermal protection, mechanical performance, and microbial viability. However, it is important to note that the current model’s predictive scope is limited to *Bacillus subtilis* spores encapsulated using CFBB and gelatin capsules with cement paste, and subjected to ISO 834 standard fire curves. While the results provide useful insights into thermal protection strategies and self-healing potential, the framework is not yet generalizable across other biological agents, encapsulation types, or fire exposure conditions.

### Key insights include

- Thermal survival modelling revealed that CFBB_CP significantly extends bacterial viability across fire scenarios. Simulations indicated survival durations of up to 19.48 h at 200 °C and 3.94 h at 800 °C, attributable to the superior thermal stability of carbon fiber (decomposition > 600 °C). Gelatin-based encapsulations, by contrast, failed to protect bacterial spores beyond 200 °C, demonstrating negligible survival times.
- The numerical model, grounded in FDM heat transfer analysis and incorporating bacterial inactivation kinetics, accurately mirrored experimental results. Thermal imaging and compressive strength retention supported the spatial survival patterns predicted by the model, particularly near the concrete core. Localized post-fire calcium carbonate precipitation further corroborated model accuracy and bacterial reactivation potential.
- CFBB_CP encapsulation conferred dual benefits—thermal insulation and structural reinforcement. Specimens retained 40–45% of original strength at 600 °C, compared to < 25% in GP_CP. CFBB_CP also demonstrated microstructural resilience post-fire, offering a robust platform for bacterial survival and self-healing activation.
- Sensitivity analyses emphasized the critical role of encapsulation geometry. Encapsulation layers of 1.75–2.0 mm delayed heat diffusion effectively, maintaining internal temperatures below lethal thresholds for up to 5 h at 600 °C. Thinner layers allowed rapid thermal ingress, drastically reducing bacterial survival time.
- Gelatin-based encapsulations, though structurally simple, proved unsuitable for fire-resilient applications due to early decomposition, void formation, and poor bonding, which accelerated crack propagation and thermal damage.

Overall, this research advances a predictive and experimentally validated design methodology for fire-resistant self-healing concrete. By linking numerical heat diffusion models with bacterial survivability and structural performance, it provides practical guidance for optimizing encapsulation strategies in high-risk environments. These results emphasize the need for encapsulation systems that provide insulation, protect bacteria, and support concrete strength during fires.

Future validation across diverse bacterial strains, encapsulation materials (e.g., mineral-based systems), and non-standard fire scenarios is essential to generalize the framework. Such efforts will enable broader application of the model in real-world structural fire environments and support the development of more resilient bio-concrete systems. Future work should also focus on:

- Developing hybrid encapsulation systems, combining CFBB with ceramic or aerogel coatings for extreme-temperature resistance (> 800 °C),
- Exploring thermophilic bacterial strains to extend the operational envelope of bio-concrete,
- Conducting full-scale fire testing on structural elements to validate scalability and performance under realistic fire loads.

By bridging predictive modelling with material engineering and microbial functionality, this study lays the groundwork for next-generation bio-concrete systems tailored for high-risk, high-performance infrastructure.

## Data Availability

All data generated or analyzed during this study are included in this published article.

## References

[1] Ahmad, F. et al. Fire resistance and thermal performance of hybrid fibre-reinforced magnesium oxychloride cement-based composites. *Constr. Build. Mater.* **472**, 140867. 10.1016/j.conbuildmat.2025.140867 (2025).

[2] Ashteyat, A., Shhabat, M., Alkhalaileh, A., Al-Zu’bi, M. & Abdel-Jaber, M. Behavior of ultra-high-performance concrete under elevated temperatures: A comprehensive review of mechanical, physical, thermal, and microstructural properties. *Results Eng.* **26**, 104960. 10.1016/j.rineng.2025.104960 (2025).

[3] Ünverdi, M., Kaya, Y., Mardani, N. & Mardani, A. Investigation of the microstructural and mechanical properties of Fiber-Reinforced Roller-Compacted concrete under High-Temperature exposure. *Materials* **18**, 2430. 10.3390/ma18112430 (2025). 40508427 10.3390/ma18112430 PMC12155776

[4] Zhao, L. et al. Mechanical properties of hybrid fibers and nano-silica reinforced concrete during exposure to elevated temperatures. *Case Stud. Constr. Mater.* **21**, e04042. 10.1016/j.cscm.2024.e04042 (2024).

[5] Pham, T. M. Fibre-reinforced concrete: State-of-the-art-review on bridging mechanism, mechanical properties, durability, and eco-economic analysis. *Case Stud. Constr. Mater.* **22**, e04574. 10.1016/j.cscm.2025.e04574 (2025).

[6] Khan, M., Kai, M., Ahmad, M. R., Lao 劳健聪, J. C. & Dai, J. G. Fire performance of Fiber-reinforced Ultra-High-Performance concrete: A state-of-the-art review. *J. Asian Concrete Federation*. **9**, 65–102. 10.18702/acf.2023.9.1.65 (2023).

[7] Amran, M., Huang, S. S., Onaizi, A. M., Murali, G. & Abdelgader, H. S. Fire spalling behavior of high-strength concrete: A critical review. *Constr. Build. Mater.* **341**, 127902. 10.1016/j.conbuildmat.2022.127902 (2022).

[8] Ding, C. et al. Research on fire resistance of silica fume insulation mortar. *J. Mater. Res. Technol.* **25**, 1273–1288. 10.1016/j.jmrt.2023.06.004 (2023).

[9] Ukpata, J. O., Ewa, D. E., Liwhuliwhe, J. U., Alaneme, G. U. & Obeten, K. E. Effects of elevated temperatures on the mechanical properties of laterized concrete. *Sci. Rep.* **13**, 18358. 10.1038/s41598-023-45591-5 (2023). 37884737 10.1038/s41598-023-45591-5 PMC10603149

[10] Liu, C. & Chen, J. High temperature degradation mechanism of concrete with plastering layer. *Materials* **15**, 398. 10.3390/ma15020398 (2022). 35057116 10.3390/ma15020398 PMC8780578

[11] Huang, J., Li, J., Shao, R. & Wu, C. Effect of high temperature and cooling method on compression and fracture properties of geopolymer-based ultra-high performance concrete. *J. Building Eng.* **105**, 112433. 10.1016/j.jobe.2025.112433 (2025).

[12] Zhang, Z. et al. Use of polypropylene fibers to mitigate spalling in high strength PE-ECC under elevated temperature. *Case Stud. Constr. Mater.* **22**, e04381. 10.1016/j.cscm.2025.e04381 (2025).

[13] Althoey, F., Ansari, W. S., Sufian, M. & Deifalla, A. F. Advancements in low-carbon concrete as a construction material for the sustainable built environment. *Developments Built Environ.* **16**, 100284. 10.1016/j.dibe.2023.100284 (2023).

[14] Luhar, S., Nicolaides, D. & Luhar, I. Fire resistance behaviour of geopolymer concrete: an overview. *Buildings* **11**, 82. 10.3390/buildings11030082 (2021).

[15] Wong, P. Y. et al. Advances in microbial self-healing concrete: A critical review of mechanisms, developments, and future directions. *Sci. Total Environ.* **947**, 174553. 10.1016/j.scitotenv.2024.174553 (2024). 38972424 10.1016/j.scitotenv.2024.174553 PMC11299504

[16] Rajadesingu, S. et al. State-of-the-art review on advancements of eco-friendly bacterial-infused self-healing concrete for sustainable constructions. *J. Building Eng.* **91**, 109669. 10.1016/j.jobe.2024.109669 (2024).

[17] Amran, M. et al. Self-Healing concrete as a prospective construction material: A review. *Mater. (Basel)*. **15**, 3214. 10.3390/ma15093214 (2022). 10.3390/ma15093214 PMC9106089 35591554

[18] Vishal, A., Chepuri, A. & Chandana, N. Assessment of bacteria-based self-healing concrete through experimental investigations — a sustainable approach. *J. Mater. Science: Mater. Eng.* **20**, 15. 10.1186/s40712-025-00215-w (2025).

[19] Zhou, W. et al. Inactivation of bacterial spores subjected to sub-second thermal stress. *Chem. Eng. J.* **279**, 578–588. 10.1016/j.cej.2015.05.021 (2015).

[20] Xiao, J., Li, Z., Xie, Q. & Shen, L. Effect of strain rate on compressive behaviour of high-strength concrete after exposure to elevated temperatures. *Fire Saf. J.* **83**, 25–37. 10.1016/j.firesaf.2016.04.006 (2016).

[21] Georgali, B. & Tsakiridis, P. E. Microstructure of fire-damaged concrete. A case study. *Cem. Concr. Compos.* **27**, 255–259. 10.1016/j.cemconcomp.2004.02.022 (2005).

[22] Buchanan, A. H. & Abu, A. K. *Structural Design for Fire Safety* (Wiley, 2017).

[23] Vedrtnam, A., Bedon, C. & Barluenga, G. Study on the compressive behaviour of sustainable Cement-Based composites under One-Hour of direct flame exposure. *Sustainability* **12**, 10548. 10.3390/su122410548 (2020).

[24] Yaqoob Wani, I. & Singh, K. Effect of encapsulated bacteria on concrete properties: A review, Materials Today: Proceedings 33 1706–1712. (2020). 10.1016/j.matpr.2020.07.540

[25] Nery, M. et al. Mechanisms of encapsulation of bacteria in self-healing concrete: review, DYNA 86 17–22. (2019). 10.15446/dyna.v86n210.75343

[26] Pungrasmi, W., Intarasoontron, J., Jongvivatsakul, P. & Likitlersuang, S. Evaluation of microencapsulation techniques for MICP bacterial spores applied in Self-Healing concrete. *Sci. Rep.* **9**, 12484. 10.1038/s41598-019-49002-6 (2019). 31462752 10.1038/s41598-019-49002-6 PMC6713760

[27] Jiang, L. et al. State-of-the-Art review of microcapsule Self-Repairing concrete: principles, applications, test methods. *Prospects Polym.* **16**, 3165. 10.3390/polym16223165 (2024). 10.3390/polym16223165 PMC11598030 39599256

[28] Yang, S. et al. Encapsulation techniques, action mechanisms, and evaluation models of probiotics: recent advances and future prospects. *Food Front.* **5**, 1212–1239. 10.1002/fft2.374 (2024).

[29] Mansouripour, S., Esfandiari, Z. & Nateghi, L. The effect of heat process on the survival and increased viability of probiotic by microencapsulation: a review. *Annals Biol. Res.* **4**, 83–87 (2013).

[30] Barroso, G., Li, Q., Bordia, R. K. & Motz, G. Polymeric and ceramic silicon-based coatings – a review. *J. Mater. Chem. A*. **7**, 1936–1963. 10.1039/C8TA09054H (2019).

[31] Owens, P. L. & Newman, J. B. 7 - Lightweight aggregate manufacture, in: (eds Newman, J. & Choo, B. S.) Advanced Concrete Technology, Butterworth-Heinemann, Oxford, : 1–12. 10.1016/B978-075065686-3/50283-4. (2003).

[32] Podnar, T. M. & Kravanja, G. Thermal, mechanical, and microstructural properties of novel light expanded clay aggregate (LECA)-Based geopolymer concretes. *J. Compos. Sci.* **9**, 69. 10.3390/jcs9020069 (2025).

[33] Yan, Y. et al. Application of expanded perlite immobilized microorganisms in cementitious materials. *J. Building Eng.* **76**, 106834. 10.1016/j.jobe.2023.106834 (2023).

[34] Vedrtnam, A. et al. Experimental and numerical study on post-fire self-healing concrete for enhanced durability. *Sci. Rep.* **15**, 9731. 10.1038/s41598-025-94331-4 (2025). 40118932 10.1038/s41598-025-94331-4 PMC11928560

[35] Cheng, P., Zhu, H., Zhang, Y., Jiao, Y. & Fish, J. Coupled thermo-hydro-mechanical-phase field modeling for fire-induced spalling in concrete. *Comput. Methods Appl. Mech. Eng.* **389**, 114327. 10.1016/j.cma.2021.114327 (2022).

[36] Kodur, V. & Banerji, S. Modeling the fire-induced spalling in concrete structures incorporating hydro-thermo-mechanical stresses. *Cem. Concr. Compos.* **117**, 103902. 10.1016/j.cemconcomp.2020.103902 (2021).

[37] Klemczak, B., Smolana, A. & Jędrzejewska, A. Modeling of heat and mass transfer in cement-Based materials during cement Hydration—A. *Rev. Energies*. **17**, 2513. 10.3390/en17112513 (2024).

[38] Banerjee, D. A review of models for heat transfer in steel and concrete members during fire. *J. Res. Natl. Inst. Stand. Technol.* **126**10.6028/jres.126.30 (2021). 10.6028/jres.126.030 PMC11249205 39081637

[39] Tahersima, M. & Tikalsky, P. Finite element modeling of hydration heat in a concrete slab-on-grade floor with limestone blended cement. *Constr. Build. Mater.* **154**, 44–50. 10.1016/j.conbuildmat.2017.07.176 (2017).

[40] Javeed, Y., Goh, Y., Mo, K. H., Yap, S. P. & Leo, B. F. Microbial self-healing in concrete: A comprehensive exploration of bacterial viability, implementation techniques, and mechanical properties. *J. Mater. Res. Technol.* **29**, 2376–2395. 10.1016/j.jmrt.2024.01.261 (2024).

[41] Banerjee, D. K. A review of models for heat transfer in steel and concrete members during fire. *J. Res. Natl. Inst. Stand. Technol.* **126**, 126030. 10.6028/jres.126.030 (2021). 39081637 10.6028/jres.126.030 PMC11249205

[42] de Silva, D., Sassi, S., De Rosa, G., Corbella, G. & Nigro, E. Effect of the fire modelling on the structural temperature evolution using advanced calculation models. *Fire* **6**, 91. 10.3390/fire6030091 (2023).

[43] Li, M. et al. Stress induced carbon fiber orientation for enhanced thermal conductivity of epoxy composites. *Compos. Part. B: Eng.* **208**, 108599. 10.1016/j.compositesb.2020.108599 (2021).

[44] Wolterbeek, T. K. T. & Hangx, S. J. T. The thermal properties of set Portland cements – a literature review in the context of CO2 injection well integrity. *Int. J. Greenhouse Gas Control*. **126**, 103909. 10.1016/j.ijggc.2023.103909 (2023).

[45] Remesar, J. C., Vera, S. & Lopez, M. Assessing and Understanding the interaction between mechanical and thermal properties in concrete for developing a structural and insulating material. *Constr. Build. Mater.* **132**, 353–364. 10.1016/j.conbuildmat.2016.11.116 (2017).

[46] Miao, Y. et al. Micro/meso-scale damage analysis of recycled aggregate concrete mixed with glazed Hollow beads after high temperatures based on 2D CT images. *Constr. Build. Mater.* **365**, 130063. 10.1016/j.conbuildmat.2022.130063 (2023).

[47] Sassine, E., Cherif, Y., Dgheim, J. & Antczak, E. Investigation of the mechanical and thermal performances of concrete Hollow blocks. *SN Appl. Sci.* **2**, 2006. 10.1007/s42452-020-03881-x (2020).

[48] Wiktor, V. & Jonkers, H. M. Quantification of crack-healing in novel bacteria-based self-healing concrete. *Cem. Concr. Compos.* **33**, 763–770. 10.1016/j.cemconcomp.2011.03.012 (2011).

[49] Vedrtnam, A., Gunwant, D., Kalauni, K. & Palou, M. T. Experimental and numerical study on sustainable post-fire repair of concrete structures using bacterial self-healing mechanisms. *Constr. Build. Mater.* **474**, 141175. 10.1016/j.conbuildmat.2025.141175 (2025).

[50] Vedrtnam, A., Kalauni, K. & Palou, M. T. Finite element simulation of bacterial self-healing in concrete using microstructural transport and precipitation modeling. *Sci. Rep.* **15**, 15809. 10.1038/s41598-025-99844-6 (2025). 40328859 10.1038/s41598-025-99844-6 PMC12056202

[51] Zhang, J., Yu, M., Chu, X. & Li, R. A coupled hygro-thermo-mechanical Cosserat peridynamic modelling of fire-induced concrete fracture. *Acta Mech.* **235**, 3799–3829. 10.1007/s00707-024-03891-5 (2024).

[52] Ding, C., Xue, K. & Yi, G. Research on fire resistance and economy of basalt fiber insulation mortar. *Sci. Rep.* **13**, 17288. 10.1038/s41598-023-44591-9 (2023). 37828256 10.1038/s41598-023-44591-9 PMC10570304

